# Melanins as Sustainable Resources for Advanced Biotechnological Applications

**DOI:** 10.1002/gch2.202000102

**Published:** 2020-11-25

**Authors:** Hanaa A. Galeb, Emma L. Wilkinson, Alison F. Stowell, Hungyen Lin, Samuel T. Murphy, Pierre L. Martin‐Hirsch, Richard L. Mort, Adam M. Taylor, John G. Hardy

**Affiliations:** ^1^ Department of Chemistry Lancaster University Lancaster LA1 4YB UK; ^2^ Department of Chemistry Science and Arts College Rabigh Campus King Abdulaziz University Jeddah 21577 Saudi Arabia; ^3^ Department of Biomedical and Life Sciences Lancaster University Lancaster LA1 4YG UK; ^4^ Department of Organisation, Work and Technology Lancaster University Management School Lancaster University Lancaster LA1 4YX UK; ^5^ Department of Engineering Lancaster University Lancaster LA1 4YW UK; ^6^ Materials Science Institute Lancaster University Lancaster LA1 4YB UK; ^7^ Lancashire Teaching Hospitals NHS Trust Royal Preston Hospital Sharoe Green Lane Preston PR2 9HT UK; ^8^ Lancaster Medical School Lancaster University Lancaster LA1 4YW UK

**Keywords:** applications, biotechnology, material science, melanin, polymers, sustainable development

## Abstract

Melanins are a class of biopolymers that are widespread in nature and have diverse origins, chemical compositions, and functions. Their chemical, electrical, optical, and paramagnetic properties offer opportunities for applications in materials science, particularly for medical and technical uses. This review focuses on the application of analytical techniques to study melanins in multidisciplinary contexts with a view to their use as sustainable resources for advanced biotechnological applications, and how these may facilitate the achievement of the United Nations Sustainable Development Goals.

## Graphical abstract

Melanin‐based materials have broad applicability in materials science and engineering for medical and technical applications. This review offers an overview of the techniques used to analyze their chemical, electrical, optical, and paramagnetic properties with a view to their use as sustainable resources for advanced biotechnological applications that may help the achieve the United Nations Sustainable Development Goals.

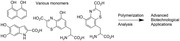

## 1 Introduction

**Figure 1:**
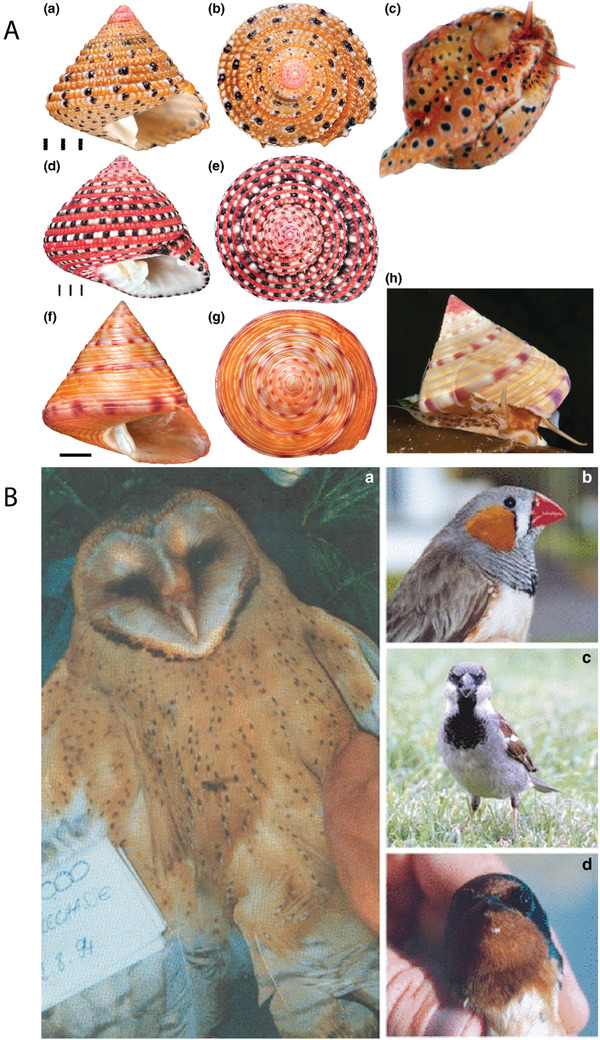
Eye catching examples of color in nature (including melanin‐derived colors). A) Photographs of colorful seashells. (a–c) *Clanculus margaritarius* C. (a,b) Two views of a shell of *Clanculus margaritarius* C (specimen #2). Note that this specimen is subadult. (c) Colored foot of a live animal. Note that the color and pattern are the same as found on the shell. (d,e) Two views of a *Clanculus pharaonius* shell (specimen #4). (f–h) *Calliostoma zizyphinum*. (f,g) Two views of a shell of *Calliostoma zizyphinum* (specimen #2). (h) Living animal showing foot color (not the same specimen). Note that the foot color and pattern in this species do not match the shell. Scale bars for *Clanculus spp* are in mm. Scale bar for *Calliostoma* is 1 cm. Reproduced with permission.^[^ ^729^ ^]^ Copyright 2017, Wiley. B) Color photos showing the melanin‐based ornamental traits in four species that have been well‐studied in the context of metals, amino acids, and hormones. (a) Black breast spotting and chestnut breast coloring in a barn owl. (b) Black breast striping and patch in a male zebra finch. (c) Black throat badge of male house sparrow. (d) Brown forehead and throat plumage in a barn swallow. Reproduced with permission.^[^ ^13^ ^]^ Copyright 2008, Wiley.

The color a light source has is dependent on the wavelengths of light mixed together. Perception of color is dependent on the species observing an object,^[^ ^1^, ^2^ ^]^ and the color of an object is dependent on the parts of the spectrum visible to the species that are not absorbed (e.g., by dyes, pigments, etc.) and reflected/scattered/transmitted, and/or interference effects.^[^ ^3^, ^4^, ^5^, ^6^, ^7^, ^8^ ^]^ Colorants (dyes and pigments) impart color to a material; dyes are molecular species, whereas pigments are particulates (and their color tends to be more stable than dyes (i.e., less likely to bleach)).^[^ ^9^ ^]^ Dyes and pigments absorb light in specific wavelength ranges due to their system of conjugated bonds in their structure (i.e., chromophore) and are responsible for some of the colors we and other species observe, as exemplified by the dark spots in seashells,^[^ ^10^, ^11^, ^12^ ^]^ and birds feathers^[^ ^13^ ^]^ (shown in **Figure** **1**).

**Figure 2:**
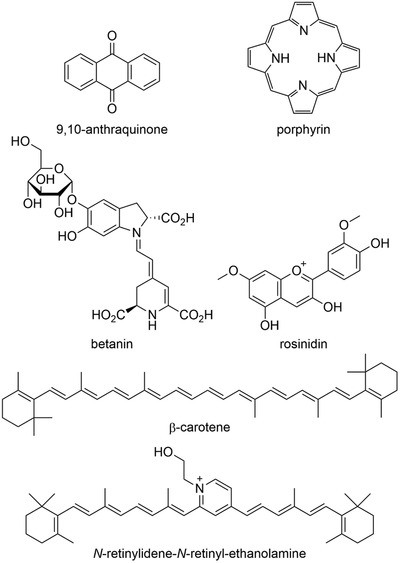
Examples of naturally occurring pigments.

Naturally occurring pigments (known as biochromes), are synthesized and accumulated in, or excreted from, living organisms (animals, bacteria, and plants). They can be classified into six major groups as *N*‐heterocyclic derivatives (e.g., betalaines (such as betanin) and eumelanins), *O*‐heterocyclic derivatives (e.g., anthocynins (such as rosinidin), and other flavonoid pigments); quinones (e.g., derivatives of anthraquinone (such as 9,10‐anthraquinone), benzoquinone, naphthoquinone, etc.); tetrapyrroles (e.g., porphyrin derivatives such as chlorophyll and heme); tetraterpenoid derivatives (e.g., carotenoids [such as β‐carotene] and iridoids); and “miscellaneous” (e.g., lipofuscins (such as *N*‐retinylidene‐*N*‐retinyl‐ethanolamine) and fungal pigments), see **Figure** **2**.^[^ ^14^, ^15^, ^16^ ^]^

**Figure 3:**
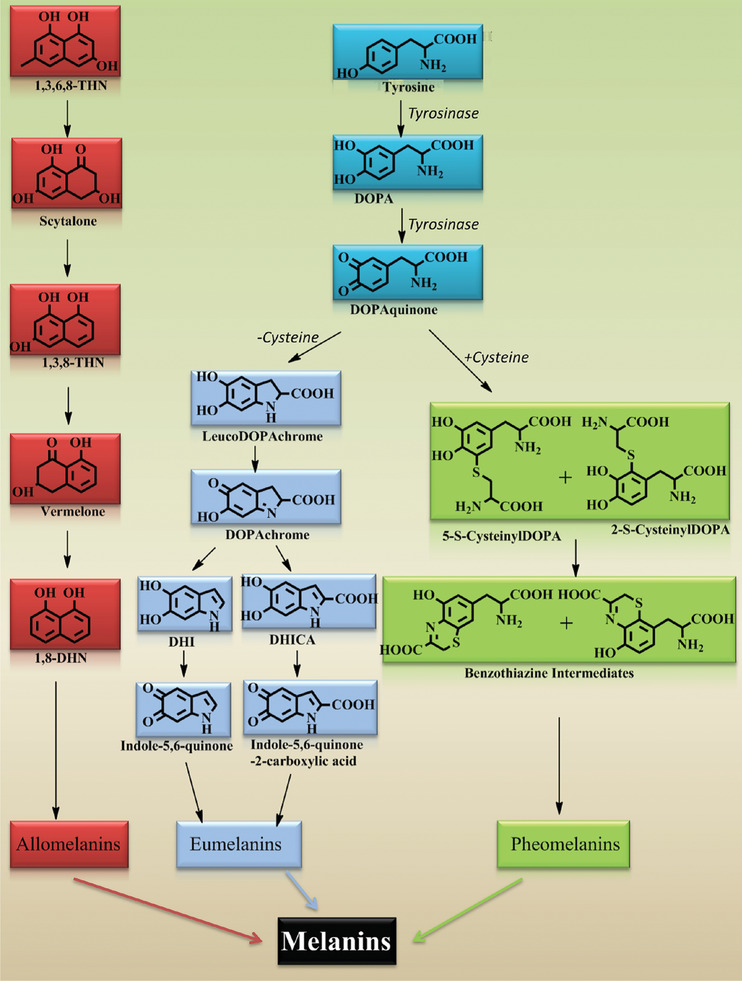
Common biosynthetic pathways for melanins. Reproduced with permission.^[^ ^47^ ^]^ Copyright 2018, American Chemical Society.

Melanins are a class of biopolymers with diverse origins and chemical compositions, which are widespread in nature, and the focus of this review (representative structures are shown in **Figure** **3**). While the name melanin was initially applied to black pigments, it has subsequently been used to describe pigments of colors from black/brown eumelanins^[^ ^17^ ^]^ to red/yellow pheomelanins,^[^ ^18^, ^19^ ^]^ all of which play a role in skin pigmentation,^[^ ^20^ ^]^ in combination with carotenoids, hemoglobin, etc.^[^ ^17^, ^21^ ^]^ Melanins have a variety of functions in nature (from photoprotection to photosensitization,^[^ ^20^, ^22^ ^]^ antioxidant defense, and metal/drug binding),^[^ ^23^, ^24^, ^25^, ^26^ ^]^ which reflect a unique combination of chemical, electrical, optical, and paramagnetic properties,^[^ ^27^, ^28^, ^29^, ^30^ ^]^ and their properties have resulted in their application in materials science for a range of historical,^[^ ^31^ ^]^ medical, and technical applications.^[^ ^32^, ^33^, ^34^, ^35^, ^36^, ^37^ ^]^

**Figure 4:**
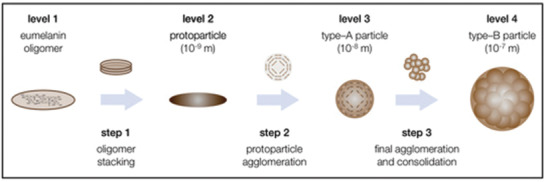
Most recent version of the three‐step, four‐level hierarchical buildup mechanism of natural and biomimetic eumelanin, based on the state of literature. Reproduced with under the terms of the CC BY license.^[^ ^49^ ^]^ Copyright 2017, MDPI.

Melanin production is typically an oxidative process involving reactive oxygen species occurring in vivo, often also involving enzymes such as oxidases (e.g., phenolases that catalyze the oxidation of phenol derivatives [eumelanins and pheomelanins are produced within melanocytes by a complex biosynthetic pathway involving the tyrosinase‐catalyzed oxidation of tyrosine]) followed by uncontrolled polymerization of the oxidized intermediates (often involving a reactive quinone intermediate prone to reactions with amine and hydroxyl groups and capable of undergoing reversible redox reactions).^[^ ^19^ ^]^ In contrast to the production of polynucleic acids (e.g., DNA, RNA) and proteins, melanin production does not involve “templates” and therefore the compositions and sequences of “monomers” in the backbone of the melanins is random (albeit clearly influenced by the feedstocks available, organism/tissue, and other conditions); however, eumelanins are rich in l‐DOPA,^[^ ^38^ ^]^ pheomelanin is rich in 5‐cys‐DOPA,^[^ ^38^ ^]^ neuromelanins are rich in 5,6‐dihydroxyindole (DHI),^[^ ^39^, ^40^ ^]^ catechol melanins are rich in catecholic monomers,^[^ ^41^, ^42^ ^]^ insect melanin is rich in *N*‐acetyl‐dopamine,^[^ ^43^, ^44^ ^]^ pyomelanin is rich in homogentisic acid (HGA), and allomelanins are rich in 1,8‐dihydroxynaphthalene (DHN), see Figure 3.^[^ ^19^, ^45^ ^]^ Oligomeric species (e.g., trichochromes occurring in hair^[^ ^46^ ^]^ tend to have relatively low molecular weights and are soluble; by comparison, the polymerization of melanins^[^ ^47^ ^]^ yields species with higher molecular weights and the formation of insoluble pigment particles (**Figure** **4**). The generation of these insoluble pigment particles^[^ ^48^ ^]^ is proposed to proceed via a nucleation and growth mechanism (Figure 4) as detailed in an excellent review by Strube and co‐workers.^[^ ^49^ ^]^ Melanins are produced by a variety of life forms including bacteria,^[^ ^50^, ^51^ ^]^ and eukarya (e.g., fungi^[^ ^46^, ^52^, ^53^, ^54^ ^]^ plants,^[^ ^55^, ^56^ ^]^ animals,^[^ ^57^, ^58^, ^59^ ^]^ and humans^[^ ^18^, ^60^ ^]^), and play a role in the conversion of radiation into chemical energy for growth, opening up the potential for them to play a critical role for life in extreme environments on Earth and perhaps elsewhere in the universe.^[^ ^61^, ^62^, ^63^, ^64^, ^65^, ^66^, ^67^, ^68^, ^69^, ^70^ ^]^

A multitude of reviews on melanins exist, covering various aspects of their biochemistry, functions, and applications.^[^ ^47^, ^57^, ^59^, ^71^, ^72^, ^73^, ^74^, ^75^, ^76^, ^77^, ^78^, ^79^, ^80^, ^81^, ^82^ ^]^ This review focuses on the application of analytical techniques to study melanins in multidisciplinary contexts with a view to their use as sustainable resources for advanced biotechnological applications. The scope of the literature prevents this from being comprehensive in coverage, however, we have attempted to ensure it covers a wide variety of analytical techniques applied to melanins, highlighting a few examples of the insights drawn from analysis of melanins^[^ ^83^ ^]^ and their precursors produced by bacteria and eukarya (including laboratory‐based scientists and engineers). We believe therefore that this may be of interest to researchers from multidisciplinary backgrounds seeking an overview of techniques used to study this class of biomolecules with a view to their novel biotechnological applications,^[^ ^84^, ^85^ ^]^ which are classified in a color coded fashion that encompasses broad areas of use (**Table** **1**).^[^ ^86^ ^]^ White biotechnologies (industrial biotechnology) involve the application of biotechnology to industrial processes (e.g., enzyme mediated synthesis, synthetic/engineering biology);^[^ ^87^ ^]^ gold biotechnologies involve computational/bioinformatics approaches;^[^ ^86^ ^]^ blue biotechnologies involve the use of marine/sea resources;^[^ ^88^, ^89^, ^90^ ^]^ green biotechnologies are related to agricultural processes;^[^ ^87^ ^]^ yellow biotechnologies are related to food production and control/use of insects;^[^ ^87^ ^]^ gray biotechnologies are related to environmental applications (e.g., maintenance of biodiversity and the remediation of pollutants);^[^ ^87^ ^]^ brown biotechnologies are related to the management of arid lands and deserts (intrinsically linked to our current interdisciplinary understanding of climate change);^[^ ^87^ ^]^ dark biotechnologies encompass bioterrorism, biological weapons, and biowarfare (e.g., microorganisms and toxins to cause diseases/death in humans, livestock, and crops);^[^ ^91^, ^92^ ^]^ red biotechnologies are for medical, pharmaceutical, and health applications;^[^ ^87^, ^93^, ^94^ ^]^ and purple/violet biotechnologies encompass ethics, laws, and philosophical issues surrounding biotechnologies.^[^ ^95–^, ^99^ ^]^ Throughout the review we highlight examples of melanins in the context of various biotechnological applications, and how these may facilitate our achievement of the United Nations Sustainable Development Goals (UN SDGs) by concomitantly engaging with the UN's Six Principles for Responsible Management Education (PRME) initiative.^[^ ^100^, ^101^, ^102^, ^103^, ^104^, ^105^, ^106^ ^]^

**Table 1:** Biotechnology types

Biotechnology classification	Scope	Reference(s)
Blue biotechnology	Marine/sea: use of marine/sea resources to create products and industrial applications.	^[^ ^88^, ^89^, ^90^ ^]^
Brown biotechnology	Management of arid lands and deserts: innovation/creation of biotechnologies to enable/manage agriculture in arid lands and deserts.	^[^ ^87^ ^]^
Dark biotechnology	Defense: biotechnology related to bioterrorism, biological weapons, and biowarfare (e.g., microorganisms and toxins to cause diseases/death in humans, livestock, and crops).	^[^ ^91^, ^92^ ^]^
Gold biotechnology	Computational/bioinformatics: in silico biotechnology for the development and production of products (e.g., compound identification and toxicity/function screening).	^[^ ^86^ ^]^
Gray biotechnology	Environmental applications: biotechnologies focused on the maintenance of biodiversity and the remediation of pollutants.	^[^ ^87^ ^]^
Green biotechnology	Agricultural: use of agricultural processes (e.g., transgenic plants) to produce feedstocks/materials.	^[^ ^87^ ^]^
Purple/violet biotechnology	Ethics/law/philosophy: issues surrounding biotechnology.	^[^ ^87^, ^93^, ^94^ ^]^
Red biotechnology	Medical technology: biotechnology for medical, pharmaceutical, and health applications.	^[^ ^87^, ^93^, ^94^ ^]^
White biotechnology	Industrial biotechnology: biotechnology applied to industrial processes (e.g., enzyme‐mediated synthesis, synthetic/engineering biology) for the development and production/processing of valuable chemicals and materials.	^[^ ^87^ ^]^
Yellow biotechnology	Food and insect: biotechnology used to produce food, or biotechnology to control/use insects.	^[^ ^87^ ^]^

## 2 Analysis of Melanins

Melanins produced by organisms have been separated and analyzed by various methods,^[^ ^107^, ^108^, ^109^, ^110^ ^]^ enabling their subsequent application for fundamental and applied science, technology, engineering, and medicine. This section of the review summarizes the techniques used to study melanins (**Table** **2**) which underpins their biotechnological applications in various industry sectors.

**Table 2:** Analytical techniques applied to melanins

Analytical technique	Practical application to melanins and materials containing melanins	Reference(s) to underpinning theory and practice
HPLC	HPLC has been shown to be particularly useful in the analysis of low molecular weight melanin precursors.	^[^ ^111^, ^112^, ^113^, ^114^ ^]^
Gel permeation chromatography (GPC) or size exclusion chromatography (SEC)	GPC/SEC has been shown to be particularly useful in the analysis of high molecular weight melanins (and the conversion of the low molecular weight species to high molecular weight melanins).	^[^ ^129^, ^130^ ^]^
Mass spectrometry (MS)	MS measures the mass‐to‐charge ratios of ionized species (molecules or fragments thereof) which are correlated to their molecular weights via time of flight (TOF) measurements. Low molecular weight species analyzed by techniques such as ESI or high molecular weight species by MALDI.	^[^ ^143^, ^144^, ^145^, ^146^ ^]^
Nuclear magnetic resonance (NMR) spectroscopy	NMR spectroscopy provides information about the chemical environments of spin active nuclei in materials, and therefore the chemical structure of melanins and their precursors (and potentially metal ions bound to such species) using either solution state or solid state NMR depending on the solubility of the samples.	^[^ ^160^, ^161^ ^]^
Electron paramagnetic resonance (EPR) or electron spin resonance (ESR) spectroscopy	EPR/ESR spectroscopy can be used to detect and identify free radicals and paramagnetic centers (e.g., organic radicals, metals, etc.). Melanins display paramagnetic character due to free radicals in their structures (e.g., semiquinone free radicals) which absorb microwaves under magnetic fields yielding spectra characteristic of the radical species present.	^[^ ^171^, ^172^, ^173^ ^]^
Atomic absorption spectroscopy (AAS) and atomic emission spectroscopy (AES)	AAS and AES measure the light absorbed/emitted by samples in the gaseous state (typically metal ions) and have been used to quantify the metal content of various melanins/materials.	^[^ ^161^, ^193^ ^]^
Chemiluminescence spectroscopy	Chemiluminescence spectroscopy enables measurement of light emitted as a result of a chemical reaction, and has been used to study the excited species formed through oxidative reactions (e.g., oxidized linoleic acid with melanin).	^[^ ^209^, ^210^ ^]^
Fluorescence spectroscopy	Fluorescence spectroscopy most often measures light emission from samples with electrons that have been excited, however, it is also possible to measure absorption for cases involving single/pairs of fluorophores, and has been used to study autofluorescence of melanin‐containing materials, metabolic activity of melanin‐producing species, etc.	^[^ ^209^, ^213^ ^]^
Infrared (IR) spectroscopy	Fourier transform IR (FTIR) spectroscopy relies on spectral differences for IR transmission (passing through samples), absorbance or reflection, where these differences enable functional group identification in melanins based on the energies of specific vibrational mode.	^[^ ^213^, ^227^ ^]^
Mössbauer spectroscopy	Mössbauer spectroscopy probes the properties of specific isotopic nuclei in different atomic environments by analyzing the resonant absorption of gamma rays, potentially interesting for the analysis of the interactions of metal ions with melanins.	^[^ ^241^ ^]^
Phosphorescence spectroscopy	Phosphorescence spectroscopy enables measurement of light emitted relatively slowly from a molecule, and can be used to study singlet oxygen phosphorescence (e.g., during the photobleaching of melanosomes).	^[^ ^209^ ^]^
Photoacoustic spectroscopy	Photoacoustic spectroscopy records the sound waves emitted by materials that absorb radiation, and can be used to study the melanin content of a variety of biological materials, and moreover for drug delivery and theranostic applications.	^[^ ^251^ ^]^
Photothermal spectroscopy	Photothermal spectroscopy enables measurement of heat evolved on absorption of radiation which has been applied to study melanins from various sources (e.g., synthetic melanins, the melanin content of skin).	^[^ ^280^ ^]^
Pump‐probe spectroscopy	Pump‐probe spectroscopy (and variants thereof) has been used to examine the primary photodynamics of melanins, and is useful for mapping the distribution of melanin in pigmented tissues and moreover enabling early diagnosis of melanoma.	^[^ ^290^, ^291^ ^]^
Raman spectroscopy	Raman spectroscopy relies on the inelastic scattering of monochromatic light to study the vibrational/rotational modes of molecules, and can be used to analyze bond/chromophore connectivity in melanins.	^[^ ^161^, ^251^, ^301^ ^]^
Terahertz time‐domain spectroscopy (THz‐TDS)	THz‐TDS is an efficient technique for the coherent generation and detection of broadband THz radiation for studying material response at THz frequencies, with exciting results for diagnostic imaging of cancers/melanomas during surgeries to assist removal.	^[^ ^322^, ^323^, ^324^ ^]^
UV–vis spectroscopy	UV–vis spectroscopy (in either absorption or reflectance modes) is routinely employed in the study of melanins (e.g., bond conjugation and connectivity).	^[^ ^213^ ^]^
X‐ray fluorescence (XRF) spectroscopy	XRF is often used for elemental/chemical analysis (e.g., assessing the concentrations of metal ions which are known to play important roles in oxidative damage of tissues containing melanins).	^[^ ^161^ ^]^
X‐ray photoelectron spectroscopy (XPS)	XPS offers insight into chemical composition (formula) and the chemical/electronic state of the elements in melanin‐containing materials.	^[^ ^161^, ^345^ ^]^
Scattering and diffraction	A variety of scattering and diffraction techniques (e.g., turbidimetry, nephelometry, SLS, DLS, XRD, SAXS, WAXS) enable elucidation of molecular weights of melanins, or the crystallinity and microstructure of melanin‐containing materials/species.	^[^ ^213^, ^346^, ^347^, ^348^, ^349^, ^350^, ^351^, ^352^, ^353^, ^354^, ^355^ ^]^
Thermal characterization	A variety of calorimetric methods exist for monitoring heat flow to study molecules in the solution and solid phase (e.g., calorimetry, TGA, DSC), thereby enabling elucidation of various processes including melanin formation kinetics, melanin processability and stability in various environments which are important when incorporating them in materials for various applications.	^[^ ^382^, ^383^, ^384^, ^385^, ^386^ ^]^
Electrical characterization	Electrochemical characterization of materials is useful in light of their interesting properties. Studies of reduction/oxidation processes and electron transfer using cyclic voltammetry (CV), electrochemical impedance spectroscopy (EIS) and dielectric spectroscopy enable the rational investigation of the protonic and electronic contributions.	^[^ ^399^, ^400^ ^]^
Visual and microscopic characterization	Photography offers a simple method of capturing evidence of color over a large scale (mm to km), consequently, photographs provide a useful initial starting point in studies of phenomena, including coloration of melanin‐containing materials/species.	^[^ ^427^, ^428^ ^]^
Scanning electron microscopy (SEM)	SEM is used to analyze particle size distributions and elemental compositions when used in combination with energy dispersive X‐ray spectroscopy (EDX/EDS).	^[^ ^386^, ^482^, ^483^ ^]^
Transmission electron microscopy (TEM)	TEM is used to analyze particle size distributions and elemental compositions when used in combination with energy dispersive X‐ray spectroscopy (EDX/EDS).	^[^ ^386^, ^482^, ^483^ ^]^
Scanning probe microscopy	SPM (e.g., profilometry, STM, AFM, etc.) uses various probes to analyze the surface of samples enabling examination of a multitude of properties of melanin‐containing materials/species (e.g., electronics, mechanics, spectroscopy, etc.).	^[^ ^501^, ^502^ ^]^
Computational studies	Chemoinformatic studies, such as atomistic simulations are used to study the structure of melanins and melanin‐containing materials; and bioinformatic studies are used to examine functional and structural genomics, transcriptomics, proteomics, metabolomics, glycomics, lipidomics, etc.	^[^ ^355^, ^531^, ^532^, ^533^, ^534^, ^535^, ^536^, ^537^ ^]^

### 2.1 High Performance Liquid Chromatography (HPLC)

HPLC enables the separation of low molecular weight species based on differences in their interactions with the adsorbent material (typically a column packed with silica, optionally derivatized with species including alkyl chains), causing different elution times for the different components enabling their separation and subsequent identification and quantification.^[^ ^111^, ^112^, ^113^, ^114^ ^]^ HPLC has been shown to be particularly useful in the analysis of low molecular weight melanin precursors,^[^ ^115^ ^]^ a few examples of which are highlighted. HPLC has been used to study the generation of HGA (from tyrosine and phenylalanine) and its excretion, auto‐oxidation, and self‐polymerization to form melanin for a variety of bacteria, including *Bacillus thuringiensis*,^[^ ^116^ ^]^ *Burkholderia cenocepacia*,^[^ ^117^ ^]^ *Escherichia coli*,^[^ ^118^ ^]^ *Shewanella algae*,^[^ ^119^ ^]^ *Shewanella colwelliana*,^[^ ^120^ ^]^ *Vibrio cholerae*,^[^ ^120^ ^]^ and *Rubrivivax benzoatilyticus JA2*,^[^ ^121^ ^]^ to name a few. Biosynthetic pathways under various conditions (e.g., anaerobic/aerobic conditions) can be studied, offering insight into the utilization of l‐phenylalanine as source of nitrogen under anaerobic/aerobic conditions but not as a carbon source, identification of key metabolites (e.g., l‐tyrosine, 4‐hydroxyphenylpyruvic acid, HGA), and enzyme activities leading to homogentisate accumulation and pyomelanin production.^[^ ^121^ ^]^ HPLC has been used in the analysis of fungal melanin production by studying fungal melanin intermediates and related metabolites.^[^ ^122^, ^123^ ^]^ In a fascinating study, HPLC enabled the elucidation of the effect gamma radiation on the growth of melanized fungi (*Cryptococcus neoformans*, *Cryptococcus sphaerospermum*, and *Wangiella dermatitidis*), demonstrating that they use the melanin they produce to convert gamma radiation into chemical energy for growth (i.e., that the fungi are radiotrophic, and grow faster when exposed to radiation; with clear potential for gray biotechnology applications).^[^ ^70^ ^]^ HPLC has been used as a semiquantitative method of HGA quantitation in the urine of patients with Alkaptonuria (AKU), offering opportunities for its use as a quick diagnostic tool for AKU,^[^ ^124^ ^]^ the effects of medications (e.g., antioxidants such as ascorbic acid) on AKU patients,^[^ ^125^ ^]^ for the detection of persons heterozygous for deficiency of HGA oxidase (i.e., red biotechnology).^[^ ^126^ ^]^ HPLC has also been used to analyze melanin degradation products from patients with melanoma^[^ ^127^ ^]^ and oligomeric species (trichochromes) from hair (i.e., red biotechnology).^[^ ^128^ ^]^

### 2.2 GPC or SEC

GPC (also known as SEC) is an analytical technique that separates polymers by size (as a function of their elution from columns filled with a porous gel).^[^ ^129^, ^130^ ^]^ GPC has been shown to be particularly useful in the analysis of high molecular weight melanins (and the conversion of the low molecular weight species to high molecular weight melanins), a few examples of which are highlighted. From a fundamental science perspective, GPC has been used to study the oxidation of monomers into melanins in the presence/absence of other species. GPC has been employed in studies demonstrating that: the polymerization of HGA to be enhanced at higher pH;^[^ ^131^ ^]^ the H_2_O_2_ mediated oxidation of phenolics (e.g., l‐DOPA) can yield light‐ or dark‐colored pigments depending on the oxidizing potential of the environment;^[^ ^132^, ^133^ ^]^ the presence of anionic polysaccharides during the polymerization of catecholamine precursors (including dopamine, epinephrine, and norepinephrine) resulted in the generation of larger melanin particles.^[^ ^134^ ^]^ With a view to more applied research, GPC has been used to follow melanin production via a white biotechnology approach,^[^ ^135^, ^136^, ^137^, ^138^, ^139^ ^]^ employing mutant strains of *Alcaligenes eutrophus* to transform tyrosine into p‐hydroxyphenylacetic acid, which is then converted to HGA, which subsequently polymerizes to form pyomelanin.^[^ ^140^ ^]^ GPC has also been used to characterize processes inhibiting melanogenesis in mouse melanoma cells in vitro and in brown guinea pigs in vivo,^[^ ^141^ ^]^ and moreover, the eumelanin produced by fungi (*Auricularia auricula*) that was subsequently used as a hepatoprotective antioxidant to treat mice with acute alcoholic liver injury (i.e., red biotechnology).^[^ ^142^ ^]^

### 2.3 MS

MS measures the mass‐to‐charge ratios of ionized species (molecules or fragments thereof) which are correlated to their molecular weights via time of flight (TOF) measurements.^[^ ^143^, ^144^, ^145^, ^146^ ^]^ The most common ionization methods are atmospheric pressure chemical ionization, chemical ionization, electron impact (EI), electrospray ionization (ESI), fast atom bombardment, field desorption/field ionization, matrix assisted laser desorption ionization (MALDI) and thermospray ionization; and the optimal ionization method is sample dependent. Various forms of MS have been used for the analysis of melanins and their precursors, a few examples of which are highlighted. In humans, urine of patients with alkaptonuria becomes dark due to the oxidation of HGA to benzoquinone acetic acid (BQA), which is a common means of diagnosis and the reason it is often known as black urine disease. A variety of different mass spectrometry techniques have been applied for the analysis of HGA and oxidation products thereof in samples of patient's bodily fluids including EI‐MS,^[^ ^147^ ^]^ gas chromatography coupled to MS (GC‐MS),^[^ ^148^, ^149^ ^]^ liquid chromatography coupled to MS (LC/TOF‐MS in ESI mode),^[^ ^150^ ^]^ which also enables studies of the binding of HGA and BQA to amyloids,^[^ ^151^ ^]^ which can potentially offer insight into the natural melanin formation process,^[^ ^152^, ^153^ ^]^ and give insight into potential therapeutic opportunities for removing the damaging pigment in this condition. More advanced MS setups have facilitated various studies, including LC–tandem mass spectrometry (LC–MS/MS in ESI mode) to quantify tyrosine and HGA in clinical trial samples to determine the efficacy and response to nitisinone in the treatment of AKU,^[^ ^154^ ^]^ mixtures of homovanillic acid, vanillylmandelic acid, orotic acid, and HGA,^[^ ^155^ ^]^ LC‐QTOF‐MS was used to evaluate the effect of nitisinone on the urinary metabolome of patients and mice with AKU,^[^ ^156^ ^]^ and the products of polymerization of tyrosine and HGA have been studied by MALDI‐TOF;^[^ ^17^, ^157^ ^]^ all of which serve to highlight the importance of MS techniques to study melanins for red biotechnology applications. An elegant study demonstrated the use of TOF‐secondary ion MS (TOF‐SIMS) for MS imaging of melanin‐containing fossil samples.^[^ ^158^, ^159^ ^]^ The presence of the DHN‐melanin (characterized by a variety of techniques including MALDI‐TOF) produced by the fungal banana pathogen *Mycosphaerella fijiensis* in banana leaves naturally infected with black Sigatoka disease was positively correlated to the disease stage (i.e., green/yellow biotechnology). Importantly, it was demonstrated that the melanin acted as a light‐activated phytotoxin that functions by the generation of singlet oxygen that damages the plant tissues,^[^ ^45^ ^]^ thereby highlighting the importance of such natural melanins for both green and dark biotechnologies.

### 2.4 NMR Spectroscopy

NMR spectroscopy provides information about the chemical environments of spin active nuclei in materials.^[^ ^160^, ^161^ ^]^ The choice of solution state or solid state NMR experiments is chosen based on the solubility of the melanins, with the possibility to use solution state NMR for precursors of melanins,^[^ ^150^, ^162^ ^]^ melanin–metal ion interactions,^[^ ^163^ ^]^ or indeed soluble melanins produced by yeast (e.g., *Yarrowia lipolytica* ^[^ ^164^ ^]^), or human derived neuromelanin,^[^ ^165^, ^166^ ^]^ whereas solid state NMR was necessary for the melanins produced by bacteria (*Rubrivivax benzoatilyticus* JA2^[^ ^121^ ^]^), yeast (*Cryptococcus neoformans* ^[^ ^167^ ^]^), cuttlefish (*Sepia officinalis* ^[^ ^163^, ^168^ ^]^), human hair,^[^ ^168^, ^169^ ^]^ and moreover for samples derived from human alkaptonuric joint tissues where spectral linewidths from strongly pigmented ochronotic tissue were considerably increased relative to non‐pigmented control indicating a marked increase in the level of molecular disorder in the collagen supported by electron microscope images (i.e., red biotechnology).^[^ ^170^ ^]^

### 2.5 EPR or ESR Spectroscopy

EPR or ESR spectroscopy can be used to detect and identify free radicals and paramagnetic centers (e.g., organic radicals, metals, etc.).^[^ ^171^, ^172^, ^173^ ^]^ Melanins display paramagnetic character due to free radicals in their structures (e.g., semiquinone free radicals) which absorb microwaves under magnetic fields yielding spectra characteristic of the radical species present. EPR/ESR spectroscopy is therefore a potent method of studying melanins, with reports of its use for fundamental biochemistry studies (e.g., melanin type,^[^ ^169^, ^174^, ^175^, ^176^, ^177^ ^]^ effect of pH^[^ ^178^, ^179^, ^180^ ^]^), characterising melanins from different species including bacteria (*Rubrivivax benzoatilyticus* JA2,^[^ ^121^ ^]^ *Vibrio natriegens*,^[^ ^181^ ^]^ *Streptomyces cyaneofuscatus* ^[^ ^182^ ^]^), yeast (*Cryptococcus neoformans* ^[^ ^167^ ^]^), mushrooms (*Inonotus hispidus* ^[^ ^183^ ^]^), black soldier flies (*Hermetia illucens* ^[^ ^184^, ^185^ ^]^), squid (*Loligo opalescens* ^[^ ^175^ ^]^), cuttlefish (*Sepia officinalis* ^[^ ^186^ ^]^), and cephalopod ink sacs from the Jurassic era.^[^ ^187^ ^]^ Ionizing irradiation changes the EPR/ESR signals of fungal melanins due to changes in the electronic structure of the melanins, which informed a fascinating study of melanized fungal cells (*Wangiella dermatitidis*, *Cryptococcus Neoformans* and *Cladosporium Sphaerospermum*) which displayed increased growth relative to nonmelanized cells after exposure to ionizing radiation.^[^ ^70^ ^]^ EPR/ESR can contribute to fundamental neuroscience by enhancing our understanding of the role of melanin and iron in the pathogenesis of oxidative damage in neuromelanin found in the substantia nigra,^[^ ^188^ ^]^ and moreover photoaging of eyes (i.e., red biotechnology).^[^ ^189^, ^190^ ^]^ EPR/ESR also have significant potential for the analysis of hair and skin,^[^ ^190^, ^191^ ^]^ as discussed as highlighted in an excellent review.^[^ ^192^ ^]^

### 2.6 AAS and AES

AAS and AES measure the light absorbed/emitted by samples in the gaseous state (typically metal ions).^[^ ^161^, ^193^ ^]^ AAS has been used to quantify the metal content of various melanins/materials, including Cd, Cu, Pb, Zn in synthetic DOPA melanin;^[^ ^194^, ^195^ ^]^ Al, Ca, Cu, Fe, K, Mg, Mn, Na, and Zn in polymerin (a melanin‐containing material recovered from olive oil mill wastewaters^[^ ^196^, ^197^ ^]^); Hg in melanin‐containing plant seed husks,^[^ ^198^ ^]^ Ag in fungi (*Cryptococcus neoformans* ^[^ ^199^ ^]^); Ag in flies (Drosophila melanogaster^[^ ^200^ ^]^); Ca, Fe, Mg, Na in the melanins from cuttlefish (*Sepia officinalis* ^[^ ^201^ ^]^). AES has been used to quantify the metal content of melanins, including Cu and Fe in melanins produced by bacteria (*actinomycetes* ^[^ ^202^ ^]^); Ca, Fe, K, Mn, S, and Zn in lichens;^[^ ^203^ ^]^ K, Mg, and Na in mouse hair;^[^ ^204^ ^]^ Ni, Zn in cat hair;^[^ ^205^, ^206^ ^]^ Al, As, Ca, Cd, Co, Cr, Cu, Fe, Hg, K, Mg, Mn, Mo, Na, Ni, Pb, Sb, Se, Sr, Ti, V, and Zn in human hair;^[^ ^207^ ^]^ and Fe in human neuromelanin.^[^ ^208^ ^]^

### 2.7 Chemiluminescence Spectroscopy

Chemiluminescence spectroscopy enables measurement of light emitted as a result of a chemical reaction,^[^ ^209^, ^210^ ^]^ and has been used to study the excited species formed through oxidative reaction of oxidized linoleic acid with melanin in vitro,^[^ ^211^, ^212^ ^]^ and this may play a role in ultraweak photon emissions from human skin of potential application for red biotechnologies.

### 2.8 Fluorescence Spectroscopy

Fluorescence spectroscopy^[^ ^209^, ^213^ ^]^ most often measures light emission from samples with electrons that have been excited, however, it is also possible to measure absorption for cases involving single/pairs of fluorophores.^[^ ^214^ ^]^ Fluorescence spectroscopy has been used for a variety of fundamental biochemistry studies,^[^ ^132^, ^215^ ^]^ for example, fluorescent dye binding assays were used to study functional amyloids that form filaments promoting eumelanin deposition (and thereby pigmentation in mammals);^[^ ^152^, ^216^, ^217^, ^218^ ^]^ analysis of metabolic activity of fungi (e.g., DHN–melanin and pyomelanin production by *Aspergillus niger* and *Agaricus bisporus*),^[^ ^219^, ^220^, ^221^ ^]^ and autofluorescence of melanins from cuttlefish (*Sepia officinalis*) and black human hair^[^ ^222^ ^]^ and other sources.^[^ ^223^, ^224^, ^225^, ^226^ ^]^

### 2.9 IR Spectroscopy

IR spectroscopy,^[^ ^213^, ^227^ ^]^ often FTIR spectroscopy relies on spectral differences for IR transmission (passing through samples), absorbance or reflection, where these differences enable functional group identification based on the energies of specific vibrational modes. FTIR is a very popular method of characterising melanins due to its simplicity, availability, and broad applicability, and has been used to characterize synthetic melanins (formed with a variety of monomers including dopamine,^[^ ^228^, ^229^ ^]^ 5‐S‐cys‐DOPA,^[^ ^230^ ^]^ DHN,^[^ ^231^ ^]^ and HGA;^[^ ^232^ ^]^ and optionally the presence of additives such as anionic polysaccharides^[^ ^134^ ^]^ to mimic the biological milieu) and natural melanins from bacteria (e.g., *Pseudomonas sp*.,^[^ ^233^ ^]^ *Pseudomonas stutzeri*,^[^ ^234^ ^]^ *Streptomyces cyaneofuscatus* ^[^ ^182^ ^]^), fungi (e.g., *Inonotus hispidus*,^[^ ^183^ ^]^ *Lasiodiplodia theobromae*,^[^ ^235^ ^]^ *Yarrowia lipolytica* ^[^ ^236^ ^]^), cuttlefish (*Sepia officinalis* ^[^ ^110^, ^237^, ^238^ ^]^) and humans (e.g., kidney and prostate stones,^[^ ^239^ ^]^ Egyptian mummies from 1500 B.C.^[^ ^240^ ^]^).

### 2.10 Mössbauer Spectroscopy

Mössbauer spectroscopy probes the properties of specific isotopic nuclei in different atomic environments by analyzing the resonant absorption of gamma rays.^[^ ^241^ ^]^ It has been used to analyze the interactions of metal ions (e.g., Fe^3+^) with melanins, in cuttlefish (*Sepia officinalis*) melanins^[^ ^242^, ^243^ ^]^ and neuromelanin in the substantia nigra due to its potential role in neurodegeneration, particularly Parkinson's disease (i.e., red biotechnology).^[^ ^242^, ^244^, ^245^, ^246^, ^247^, ^248^, ^249^, ^250^ ^]^

### 2.11 Phosphorescence Spectroscopy

Phosphorescence spectroscopy^[^ ^209^ ^]^ enables measurement of light emitted relatively slowly from a molecule, and has been used to study singlet oxygen phosphorescence during the photobleaching of melanosomes in vitro,^[^ ^189^ ^]^ which is important for photoaging of retinal pigments (i.e., red biotechnology).

### 2.12 Photoacoustic Spectroscopy

Photoacoustic spectroscopy records the sound waves emitted by materials that absorb radiation,^[^ ^251^ ^]^ and can be used to study a variety of biological materials,^[^ ^252^, ^253^, ^254^ ^]^ including fundamental studies,^[^ ^255^, ^256^, ^257^, ^258^ ^]^ and applied studies of the melanin content of fungi,^[^ ^259^ ^]^ cardiac muscle tissue,^[^ ^260^ ^]^ human hairs,^[^ ^261^ ^]^ tumors^[^ ^262^, ^263^, ^264^, ^265^, ^266^ ^]^ (even at the level of single cells^[^ ^267^ ^]^) and skin.^[^ ^268^, ^269^, ^270^, ^271^ ^]^ A study demonstrating the potential of photoacoustic spectroscopy to study transdermal drug delivery systems for the treatment of the pigment disorder vitiligo,^[^ ^272^ ^]^ and photoacoustic/optoacoustic imaging^[^ ^273^, ^274^ ^]^ has potential for significant medical breakthroughs (i.e., red biotechnology) potentially enabled by melanin‐based optoacoustic theranostics.^[^ ^275^, ^276^, ^277^, ^278^, ^279^ ^]^

### 2.13 Photothermal Spectroscopy

**Figure 5:**
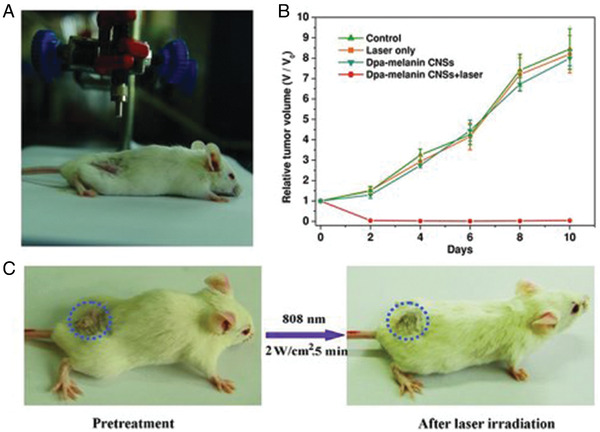
A) Photothermal therapy set‐up showing laser and the 4T1 tumor‐bearing mouse. B) Time‐dependent tumor growth curves of the mice after different treatments. C) Digital photos of a 4T1 tumor‐bearing mouse before and after photothermal therapy. Reproduced with permission.^[^ ^286^ ^]^ Copyright 2012, Wiley.

Photothermal spectroscopy^[^ ^280^ ^]^ enables measurement of heat evolved on absorption of radiation which has been applied to study melanins from various sources,^[^ ^281^, ^282^ ^]^ including synthetic melanins,^[^ ^283^ ^]^ and the melanin content of skin,^[^ ^270^, ^284^ ^]^ which can be at subcellular resolution and of use for studies of skin cancer^[^ ^285^ ^]^ and potentially treatment thereof (i.e., red biotechnology, illustrated by **Figure** **5** ^[^ ^286^ ^]^).^[^ ^7^, ^286^, ^287^, ^288^, ^289^ ^]^

### 2.14 Pump‐Probe Spectroscopy

Optical pump‐probe spectroscopy^[^ ^290^, ^291^ ^]^ has been used to examine the primary photodynamics of pheomelanin (synthetic)^[^ ^292^, ^293^, ^294^ ^]^ and eumelanin (from cuttlefish, *Sepia officinalis*,^[^ ^292^, ^293^, ^295^ ^]^ albeit with potential complications due to metal ions.^[^ ^296^ ^]^ Developments of optical pump‐probe spectroscopy include pump‐probe optical coherence microscopy that offers strong contrast between the melanotic and amelanotic regions of a nodular melanoma in human skin (potentially enabling early diagnosis of melanoma and the mapping of tumor margins during excision),^[^ ^297^ ^]^ and a multiphoton technique capable of determining the distribution of eumelanin and pheomelanin in pigmented lesions of human skin, thereby enabling differentiation of nonmalignant nevi and melanoma,^[^ ^298^, ^299^ ^]^ which is also possible at subcellular resolution,^[^ ^300^ ^]^ and highlighting the potential analytical studies of melanins for red biotechnologies.

### 2.15 Raman Spectroscopy

Raman spectroscopy^[^ ^161^, ^251^, ^301^ ^]^ relies on the inelastic scattering of monochromatic light to study the vibrational/rotational modes of molecules, and is well suited to the characterization of a variety of biological materials.^[^ ^302^ ^]^ Raman spectroscopy and variants thereof have been used to analyze bond/chromophore connectivity in eumelanin,^[^ ^303^ ^]^ cuttlefish melanins used as pigments in works of art,^[^ ^304^ ^]^ to quantify the proportions of the constituent monomers (DHI and 5,6‐dihydroxyindole‐2‐carboxylic acid (DHICA)) in eumelanins in bird feathers,^[^ ^305^ ^]^ and the molecular vibrations of pheomelanins in bird feathers have been demonstrated to be associated with reactive oxygen species production in the mitochondria of melanocytes and systemic oxidative stress and damage, potentially linking pheomelanin synthesis to human melanoma risk.^[^ ^306^ ^]^ A combination of HPLC, Raman, and computational studies demonstrated that the vibrational properties of melanins play a more significant role in the color of bird feathers than concentration‐based effects;^[^ ^307^ ^]^ moreover, a combination of Raman data and computational data demonstrate that the black color of hairs and other parts of spiders is due to eumelanin, rather than the previously suggested ommochrome Ommin A,^[^ ^308^ ^]^ thereby highlighting the potential of gold biotechnology approaches for fundamental biological studies. Raman spectroscopic analysis of the fungal melanins produced by *Neocatenulostroma genus* sustained by the colonization of gypsum in the Atacama desert (one of the driest regions of earth)^[^ ^309^ ^]^ highlights the importance of fundamental analytical studies of melanins for both brown and gray biotechnologies. The presence of eumelanins and pheomelanins in human skin offer a potentially useful clinical method for noninvasively investigating the eyes^[^ ^310^, ^311^ ^]^ or the skin.^[^ ^312^, ^313^ ^]^ Indeed, the use of Raman spectroscopy to identify melanin within cells or tissues^[^ ^314^, ^315^, ^316^, ^317^ ^]^ is becoming more routine in biomedical fields as it is emerging that differences between normal and cancer cells can be detected^[^ ^318^, ^319^, ^320^, ^321^ ^]^ and as a tool to predict cancer cells response to various therapies^[^ ^318^ ^]^ potentially allowing patients to receive specific treatments more likely to work for them as current treatment regimes are standardized with patients following on from one failed therapy to another based on the therapies generalized success rate; thereby highlighting the importance of fundamental analytical studies of melanins for red biotechnologies.

### 2.16 THz‐TDS

THz‐TDS is an efficient technique for the coherent generation and detection of broadband THz radiation for studying material response at THz frequencies.^[^ ^322^, ^323^, ^324^ ^]^ THz‐TDS has been used to investigate a variety of biological systems, with exciting results particularly in imaging. Risks associated with THz irradiation are kept to a minimum due to a relatively long wavelength thus resulting in low ionization energy. For example, at 1 THz, the photon energy is 4.1 meV, 1000 times less than what would be required for ionization. Further, the power levels used in most state‐of‐the‐art THz‐TDS systems are at the order of 10s uW, which is a hundred thousand times less than the THz radiation naturally emitted by the human body (1 W). Thermal heating effects are therefore negligible. This portion of the electromagnetic spectrum is strongly absorbed by liquid water^[^ ^325^ ^]^ due to hydrogen bonds and coincides with fundamental physical processes such as intermolecular vibrations, molecular rotational transitions, and phonon modes, which appear as resonances at THz frequencies. These features in turn underpin the contrast mechanism exploited in THz imaging/tomography and motivating clinical applications.^[^ ^237^, ^238^, ^326^ ^]^ THz‐TDS has been used to analyze the melanin content of cuttlefish ink,^[^ ^330^ ^]^ human skin equivalents with varying melanin contents^[^ ^331^, ^332^ ^]^ and thereafter skin in vivo.^[^ ^333^ ^]^ In silico modeling have enabled the generation of realistic representations of absorption and reflection of in vivo measurements.^[^ ^334^ ^]^ Even though the penetration depth of THz radiation into tissues varies according to the preparations taken and the remaining hydration (e.g., ≈0.3 mm at body temperature), additional processing steps such as applying THz penetration‐enhancing agent such as biocompatible glycerol^[^ ^335^ ^]^ or freezing, which can increase penetration depth to ≈5 mm. This may be useful for diagnostic imaging of cancers^[^ ^336^, ^337^, ^338^ ^]^ melanomas during surgeries to assist removal^[^ ^338^, ^339^ ^]^ where THz imaging has already shown significant promise (i.e., red biotechnology).^[^ ^326^ ^]^

### 2.17 UV–Vis Spectroscopy

UV–vis spectroscopy^[^ ^213^ ^]^ (in either absorption or reflectance modes) is routinely employed in the study of melanins (e.g., alkaptonuria‐derived pyomelanins) and is useful for fundamental biochemistry,^[^ ^131^, ^132^, ^133^, ^134^, ^340^ ^]^ diagnostic testing^[^ ^17^, ^150^, ^341^, ^342^ ^]^ and archaeology (e.g., Egyptian mummies^[^ ^240^ ^]^). UV–vis can be used to follow melanin production by bacteria (e.g., *Alcaligenes eutrophus*,^[^ ^140^ ^]^ *Pseudomonas sp*.,^[^ ^233^, ^343^ ^]^ *Rubrivivax benzoatilyticus*,^[^ ^121^ ^]^ *Streptomyces cyaneofuscatus* ^[^ ^182^ ^]^), and mushrooms (*Inonotus hispidus* ^[^ ^183^ ^]^), which is potentially useful for the production of melanins via a white biotechnology approach.^[^ ^135^, ^136^, ^137^, ^138^, ^139^ ^]^ The melanins produced by *Yarrowia lipolytica* yeast sequester heavy metal ions due to the presence of the metal chelating phenolics (l‐tyrosine or l‐DOPA) incorporated during its synthesis, enabling the subsequent generation of metallic nanostructures, of which, silver nanostructures were shown to displayed antifungal activity toward *Aspergillus sp*., offering potential as antifungal additives in various materials.^[^ ^236^ ^]^

### 2.18 XRF Spectroscopy

Irradiating samples with high‐energy X‐rays or gamma rays may result in the emission of fluorescent X‐rays, and XRF is often used for elemental/chemical analysis.^[^ ^161^ ^]^ This is insightful for fundamental studies of the concentrations of metal ions which are known to play important roles in oxidative damage of tissues,^[^ ^188^ ^]^ and moreover, XRF microscopy of highlighted increases in Na, Al, and Fe content and diminution of Mg content, of the tissues of patients with alkaptonuria;^[^ ^344^ ^]^ and for studies of an applied nature to examine the metal ion content (particularly Ca and Fe) of cuttlefish (*Sepia officinalis*) melanins used as pigments in works of art.^[^ ^304^ ^]^

### 2.19 XPS

XPS involves irradiating samples with X‐rays and measuring the kinetic energy and number of electrons emitted, yielding spectra that offer insight into chemical composition (formula) and the chemical/electronic state of the elements^[^ ^161^, ^345^ ^]^ XPS has been applied for a variety of different melanins and reasons, showing the presence of eumelanin in cephalopod ink sacs from the Jurassic era (>160 million years ago), highlighting the potential for fundamental science offering information to archaeological studies^[^ ^187^ ^]^ and in combination with computational studies for technological applications.^[^ ^162^ ^]^ XPS can be used to characterize and analyze the metal ion interactions with melanins, for example, metal binding by melanins produced by *Pseudomonas stutzeri*,^[^ ^234^ ^]^ highlighting the potential of such natural melanins for environmental remediation and thereby both blue and gray biotechnologies. XPS has been used to study the discoloration of a model Rattan crop (*Daemonorops margaritae*) which is cultivated on a large scale in Southeast Asia, however, discoloration diminishes its economic value, and this was shown to be primarily due to the melanins produced by fungi that grow on the rattan (in this case by XPS analysis of rattan inoculated with *Lasiodiplodia theobromae*),^[^ ^235^ ^]^ which is important for green biotechnology supported economies.

### 2.20 Scattering and Diffraction

A variety of scattering and diffraction techniques enable elucidation of the crystallinity and microstructure of materials,^[^ ^346^, ^347^, ^348^, ^349^, ^350^, ^351^, ^352^, ^353^, ^354^, ^355^ ^]^ a few examples of which will be highlighted. Turbidimetry and nephelometry are routinely used to assess growth curves of bacteria/yeast which are of potential importance for the production of melanins via a white biotechnology approach.^[^ ^356^, ^357^, ^358^, ^359^, ^360^ ^]^ Static light scattering (SLS) observes the average scattering intensity of a solution/suspension over a period of time, whereas dynamic light scattering (DLS) observes fluctuations of the scattered light over very short periods of time, offering insights into molecular weights of polymers and particle sizes (typically nanometer scale and upward). Light scattering has been used to study synthetic melanins (e.g., polydopamine^[^ ^361^, ^362^ ^]^), naturally occurring melanins in bacteria (*Vibrio natriegens* ^[^ ^181^ ^]^), yeast (*Cryptococcus neoformans* ^[^ ^167^ ^]^), fungi (*Aspergillus oryzae* ^[^ ^363^ ^]^), mushrooms (*Inonotus hispidus* ^[^ ^183^ ^]^), cuttlefish (*Sepia officinalis* ^[^ ^362^ ^]^), and can be used to study the health of eyes for patients with various conditions (e.g., for patients with pigmentary dispersion glaucoma^[^ ^364^ ^]^).

X‐ray diffraction (XRD) studies enable elucidation of the crystallinity of materials (e.g., identification/quantitation of specific phases, and/or orientation) and are suited to well‐ordered crystalline materials. Melanins tend to be noncrystalline amorphous solids due to the irregular nature of their monomer composition, consequently XRD patterns of melanins are typically broad peaks that can be relatively uninformative, exemplified by melanins produced by bacteria (e.g., *Bacillus safensis*,^[^ ^365^ ^]^ Klebsiella sp. GSK,^[^ ^366^ ^]^ *Rubrivivax benzoatilyticus* JA2,^[^ ^121^ ^]^ *Pseudomonas stutzeri* ^[^ ^367^ ^]^), yeast (*Cryptococcus neoformans*, *Aspergillus niger*, *Wangiella dermatitides*, and *Coprinus comatus* ^[^ ^368^ ^]^), frogs (*Rana esculenta* L^[^ ^369^ ^]^), cuttlefish (*Sepia officinalis* ^[^ ^370^ ^]^), and humans.^[^ ^371^, ^372^ ^]^

X‐ray scattering studies enable elucidation of the crystallinity of materials (e.g., identification/quantitation of specific phases, orientation of phases, and electron density) and are suited to non‐/semicrystalline materials. X‐ray scattering studies are classified as either small angle X‐ray scattering (SAXS) or wide angle X‐ray scattering (WAXS) depending on the distance from the sample to the detector (for WAXS the sample to detector distance is shorter and therefore diffraction maxima at larger angles are observed).^[^ ^373^ ^]^ SAXS and WAXS offer insight into the assembly of the polymer chains in a variety of melanins, which is useful from a fundamental perspective with synthetic melanins, particularly when studying the melanin assembly process from individual chains to stacks of the chains (3.4 Å spacing), that assemble into 6‐ to 10 nm sized melanin protomolecules (interacting via solvophobic and hydrogen bonding interactions);^[^ ^374^, ^375^, ^376^ ^]^ or indeed, the potential role of metal ions on the assembly process.^[^ ^377^ ^]^ A combination of SAXS and WAXS (SWAXS) has also been used to interrogate composites incorporating melanin and synthetic polymers,^[^ ^378^ ^]^ and SAXS has also been used to study natural composites (hair) revealing subtle differences in the hair of humans without/with the pigmentation disorder Alopecia Aretea, with notably smaller melanin particles in the hair of patients with Alopecia Aretea.^[^ ^379^ ^]^ Small angle neutron scattering (SANS) is complementary to SAXS because neutrons interact with atomic nuclei, whereas X‐rays interact with electron clouds, consequently neutrons penetrate matter more deeply. SANS has been used to investigate synthetic melanins based on tyrosine and the potential role of metal ions on the melanin assembly process,^[^ ^377^ ^]^ or DHI^[^ ^380^ ^]^ and the potential role of biomolecules in the DHI‐derived pigment assembly process (i.e., red biotechnology).^[^ ^381^ ^]^

### 2.21 Thermal Characterization

A variety of calorimetric methods exist for monitoring heat flow to study molecules in the solution and solid phase, thereby enabling elucidation of various processes.^[^ ^382^, ^383^, ^384^, ^385^, ^386^ ^]^ Isothermal microcalorimetry of solutions/suspensions of melanins has been used to study synthetic melanin formation kinetics (e.g., tyrosine conversion to l‐DOPA then melanin catalyzed by tyrosinase^[^ ^387^ ^]^), and the interaction of melanins with biomolecules (e.g., DNA^[^ ^388^ ^]^) or metal ions.^[^ ^389^ ^]^ Thermogravimetric analysis (TGA) and differential scanning calorimetry (DSC) are popular methods of analyzing polymer‐based materials in the solid state. TGA records differences in the mass of substances as a function of temperature or time (highlighting processes including phase transitions, absorption, desorption, chemisorptions, decomposition, etc.), whereas DSC examines how a sample's heat capacity (Cp) is changed by temperature (e.g., during transitions including melting, glass transitions, phase changes, etc.), and such data can be correlated with data obtained from scattering and diffraction experiments. The information obtained from TGA and DSC offers insight into polymer processability and stability in various environments which are important when incorporating them in materials for various applications. TGA has been used to study melanins produced by bacteria (e.g., *Klebsiella sp*. GSK^[^ ^390^ ^]^), fungi,^[^ ^391^ ^]^ garlic,^[^ ^392^ ^]^ cuttlefish (*Sepia officinalis* ^[^ ^392^, ^393^ ^]^), banana peel, and bovine eyes;^[^ ^394^ ^]^ and DSC has been used to study melanins produced by bacteria (e.g., *Pseudomonas sp*.^[^ ^395^ ^]^), fungi,^[^ ^396^ ^]^ and cuttlefish (*Sepia officinalis* ^[^ ^397^ ^]^). A study of healthy or alkaptonuric cartilage tissues used TGA and DSC to demonstrate that the total water content in healthy cartilage was higher than in AKU cartilage, that the percentage of freezable water was higher in AKU compared to healthy cartilage, and accordingly, nonfreezable water was lower in AKU compared to the control; a significant difference was observed in the heat capacity of samples, with healthy tissue showing capacity value fivefold higher. Together, the data suggest that the presence of ochronosis affects the physicochemical, thermal, and mechanical properties of the cartilage which will affect cartilage degradation (i.e., red biotechnology).^[^ ^398^ ^]^

### 2.22 Electrical Characterization

**Figure 6:**
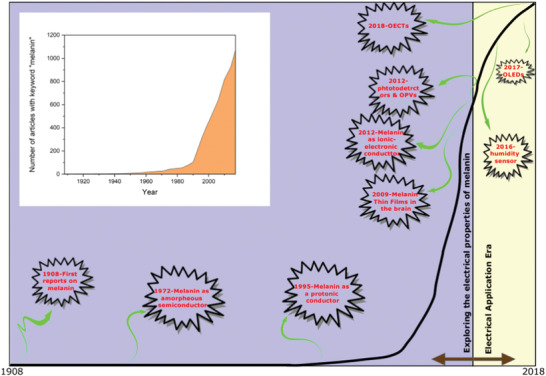
A brief timeline of melanin‐based electronics research. The black line represents the number of publication with the keyword “melanin” and key milestones in melanin‐based electronics research. Reproduced with permission.^[^ ^426^ ^]^ Copyright 2018, Elsevier B. V.

Electrical characterization of melanins and materials containing melanins is useful in light of their potential applications.^[^ ^399^, ^400^ ^]^ Studies of reduction/oxidation processes and electron transfer using cyclic voltammetry are particularly useful for melanins, with fundamental studies on melanins formed chemically from single monomers (e.g., l‐DOPA,^[^ ^401^ ^]^ DHI,^[^ ^402^ ^]^ 3,4‐dihydroxyphenylacetic acid,^[^ ^403^ ^]^ HGA,^[^ ^404^, ^405^ ^]^ DHN^[^ ^231^ ^]^), combinations of DHI and DHICA,^[^ ^406^, ^407^, ^408^ ^]^ and natural melanins from bacteria (e.g., *Shewanella oneidensis* MR‐1,^[^ ^157^ ^]^ *Pseudomonas aeruginosa* ^[^ ^409^ ^]^), plants (including fungi: basidial fungi,^[^ ^410^ ^]^ *Cryptococcus neoformans* ^[^ ^411^ ^]^ and *Nigella sativa* ^[^ ^412^ ^]^), cuttlefish (*Sepia officinalis* ^[^ ^407^ ^]^), and human hair‐derived pheomelanins.^[^ ^413^ ^]^ Electrochemical impedance spectroscopy and dielectric spectroscopy enabled the rational investigation of the protonic and electronic contributions, suggesting melanins are protonic conductors,^[^ ^414^, ^415^, ^416^ ^]^ which is important because the electrical properties of melanins^[^ ^417^, ^418^, ^419^, ^420^, ^421^, ^422^ ^]^ underpin their potential technical and medical applications,^[^ ^423^, ^424^, ^425^ ^]^ and it is noteworthy that the potential for melanins in electronics has seen an explosion of interest (see **Figure** **6** ^[^ ^426^ ^]^).

### 2.23 Visual and Microscopic Characterization

A variety of visual and microscopic techniques can be employed to study melanins and materials containing melanins.^[^ ^427^, ^428^ ^]^ Photography offers a simple method of capturing evidence of color over a large scale (mm to km), consequently, photographs provide a useful initial starting point in studies of phenomena, including architectural coloration,^[^ ^62^ ^]^ birds plumage,^[^ ^429^ ^]^ and medical case reports of gross anatomical observations of the discoloration of tissues for patients with alkaptonuria or their production of darkly colored urine.^[^ ^430^, ^431^, ^432^, ^433^, ^434^ ^]^ However, photographs do not normally differentiate the source of coloration (pigmentation, reflection, scattering, transmission, and/or interference effects^[^ ^429^, ^435^, ^436^, ^437^ ^]^) motivating the application of high‐resolution microscopy potentially combined with another analytical technique (e.g., scanning electron microscopy and energy dispersive X‐ray spectroscopy). An exception to this utilizes recent advances in digital camera technologies (potentially with smart phones) that enable hyperspectral imaging which facilitates characterization of melanins,^[^ ^438^ ^]^ and points toward some potentially very exciting developments in affordable personalized medicine that are aligned with the UN SDGs (specifically SDG 3, good health and well‐being, due to the prevalence of smart phones worldwide). Other techniques that also exploit the visible portion of the electromagnetic spectrum include reflectance colorimetry/spectrophotometry,^[^ ^439^, ^440^, ^441^, ^442^ ^]^ diffuse reflectance spectroscopy,^[^ ^443^ ^]^ and remission spectroscopy,^[^ ^444^, ^445^ ^]^ where melanin concentration on skin can be quantified via color systems such as RGB and CIELAB.

Histological studies of the microscopic anatomy of cells and tissues (of samples of in vitro, ex vivo, and in vivo studies) can be obtained using various forms of microscopy (optical, electron, scanning probe, etc.). The use of microscopy and stains to enhance pigmentation to the naked eye is important as clinically, in connective tissue disorders such as AKU, by the time the deposition of melanin like polymers in tissues occurs and is visible to the naked eye the structural and biochemical integrity of the tissue is gone. Enhancing the pigmentation process through chemical reaction in vitro^[^ ^446^, ^447^ ^]^ gives a greater indication of the origin of the pigmentation processes both intracellularly and extracellularly and represents the time at which most therapeutic interventions should be targeted. A variety of techniques have been applied to analyze samples ex vivo including: optical microscopy bone/cartilage of AKU patients;^[^ ^448^, ^449^ ^]^ two photon microscopy has been used to examine melanin from cuttlefish (*Sepia officinalis* ^[^ ^450^ ^]^); two photon fluorescence TPF microscopy^[^ ^451^ ^]^ has been used to study slices of healthy and unhealthy tissues (cartilage) of humans with alkaptonuria, observing differences in extracellular matrix density in cartilage with alkaptonuria compared with healthy cartilage; fluorescence microscopy has been used to investigate the autofluorescence properties of histologic sections of mouse eyes;^[^ ^452^ ^]^ apertureless scanning near‐field optical microscopy (capable of generating images with resolution better than the diffraction limit^[^ ^453^ ^]^) and confocal laser scanning microscopy data has been applied to analyze the distribution of melanins within zebrafish retinal tissues.^[^ ^454^ ^]^

Studies in vivo can employ optical coherence tomography (OCT) which uses low‐coherence light to capture micrometer‐resolution images within optical scattering media (and variations of OCT). It is possible to visualize melanin in skin^[^ ^455^ ^]^ and the retinal pigment epithelium via photoacoustic tomography,^[^ ^456^, ^457^, ^458^ ^]^ photothermal OCT,^[^ ^459^ ^]^ or polarization sensitive OCT^[^ ^460^, ^461^, ^462^ ^]^ if the concentration of melanin is sufficiently high, and an elegant study utilized hyperspectral OCT^[^ ^463^ ^]^ for the visualization of tissues containing significantly lower concentrations of melanin.^[^ ^464^ ^]^ It should be noted that optical methods including OCT are prone to scattering by different colorant, material compositions/structure thus leading to limited penetration depth (typically 100 µm–1 mm).^[^ ^465^, ^466^ ^]^ The optical properties of the skin can also be quantified macroscopically using spatial frequency domain spectroscopy (SFDS, which is based on diffuse optical spectroscopy) in vivo. In SFDS, tissues are illuminated with structured projections from a spatial light modulator, such as a digital micromirror device. By exploiting appropriate models of light propagation over visible to near‐infrared wavelengths, tissue absorption and scattering coefficients and chromophore concentrations can be determined in vivo.^[^ ^467^, ^468^, ^469^ ^]^ Furthermore, by combining it with techniques such as multiphoton microscopy, it is possible to obtain detailed microscopic structural information at the cellular spatial resolution thus allowing the upper dermis to be imaged. It can further provide quantitative information on the epidermis and dermis, extending the penetration depth up to ≈5 mm.^[^ ^467^, ^468^, ^469^ ^]^ Complementary computational studies of light‐tissue interaction have been used to assess melanin concentration/distributions in various organisms/tissues^[^ ^470^, ^471^, ^472^, ^473^, ^474^, ^475^, ^476^, ^477^ ^]^ and potentially adverse effects of such irradiation,^[^ ^478^, ^479^, ^480^, ^481^ ^]^ offering insight into both fundamental and applied biomedical studies (i.e., red biotechnology).

### 2.24 SEM

SEM uses a beam of electrons to illuminate samples and creates images from measurements of electrons that are reflected or back scattered off the surface of the sample, enabling analysis of particle size distributions and elemental compositions when used in combination with energy dispersive X‐ray spectroscopy (EDX/EDS).^[^ ^386^, ^482^, ^483^ ^]^ SEM and optionally EDX/EDS have been applied to study synthetic melanins,^[^ ^29^, ^231^ ^]^ and melanins produced by a variety of species, including: bacteria (*Pseudomonas sp*.,^[^ ^233^ ^]^ *Pseudomonas stutzeri*,^[^ ^234^ ^]^ *Rubrivivax benzoatilyticus* ^[^ ^121^ ^]^); yeast (*Yarrowia lipolytica* ^[^ ^236^ ^]^); fungi (*Aspergillus fumigatus*,^[^ ^484^ ^]^ *Inonotus hispidus*,^[^ ^183^ ^]^ *Mycosphaerella fijiensis*,^[^ ^45^ ^]^ *Armillaria cepistipes* ^[^ ^391^ ^]^); cuttlefish (*Sepia officinalis* ^[^ ^485^ ^]^); zebrafish,^[^ ^486^ ^]^ melanosomes isolated from human hair^[^ ^379^, ^487^ ^]^) and from bovine/fish eyes;^[^ ^488^, ^489^ ^]^ the cartilage of AKU patients,^[^ ^448^, ^451^ ^]^ and neuromelanin in the subtantia nigra of human brain tissue;^[^ ^490^ ^]^ typically observing nanometer scale particles that have aggregated to form larger particles with sizes between tens to hundreds of micrometers.

SEMs equipped with EDX/EDS have been used to quantify the elemental composition of various melanins/materials including: C and S in fossils;^[^ ^489^ ^]^ C, Ca, Cu, and O in melanins produced by bacteria (e.g., *Pseudomonas sp*.,^[^ ^233^ ^]^ *Myxococcus xanthus* and *Sinorhizobium meliloti* ^[^ ^491^ ^]^); C, K, N, Na, O, S in melanins produced by various strains of fungi (;^[^ ^391^ ^]^C, Ca, Cl, K, Mg, N, Na, O, and S in melanins produced by cuttlefish (*Sepia officinalis* ^[^ ^485^ ^]^); C, Cu, Fe, N, Na, O, S, and Zn in melanins sourced from human hair,^[^ ^379^, ^487^, ^492^ ^]^ and healthy/diseased tissues (including aortic valves, bones, brain, and cartilage containing C, O, N, S, Na, and Ca^[^ ^490^, ^493^, ^494^, ^495^ ^]^).

### 2.25 TEM

TEM uses a beam of electrons to illuminate samples and creates images from measurements of electrons that pass through very thin specimens (and contrast in images caused by differences in electron densities within different regions of the samples).^[^ ^386^, ^482^, ^483^ ^]^ TEM has been used to examine melanins and materials containing melanins from a variety of sources including: synthetic melanins (based on DHICA/DHI,^[^ ^496^ ^]^ l‐DOPA,^[^ ^236^ ^]^ DHN^[^ ^231^ ^]^); bacteria (*Pseudomonas maltophilia*,^[^ ^497^ ^]^ *Pseudomonas stutzeri*,^[^ ^234^ ^]^ *Vibrio natriegens* ^[^ ^181^ ^]^), yeast (Cryptococcus neoformans^[^ ^167^ ^]^), fungi (*Aspergillus fumigatus*,^[^ ^484^ ^]^ *Gaeumannomyces graminis var. graminis* ^[^ ^498^ ^]^), human bone osteosarcoma cell lines in vitro^[^ ^446^ ^]^ and human tissues ex vivo (e.g., aortic valves,^[^ ^493^ ^]^ bone,^[^ ^344^, ^494^ ^]^ cartilage^[^ ^170^, ^499^ ^]^); and TEM equipped with EDX/EDS has been used to quantify the elemental composition of Al, C, Ca, Cl, Cu, Fe, O, P, Si, and Zn in human melanosomes in the eye ex vivo.^[^ ^500^ ^]^

### 2.26 SPM

SPM uses various probes to analyze the surface of samples enabling examination of a multitude of properties.^[^ ^501^, ^502^ ^]^ The simplest form of SPM is contact profilometry that has been used to analyze the nanometer scale features of films of synthetic melanins (e.g., based on DHI^[^ ^503^ ^]^ and other monomers^[^ ^504^, ^505^ ^]^). Scanning tunneling microscopy (STM) is a form of scanning probe microscopy^[^ ^506^ ^]^ capable of generating high resolution images (down to the Å scale) based on quantum tunneling of electrons between the surface and the STM tip. STM has been employed to analyze synthetic melanins (e.g., based on tyrosine,^[^ ^507^, ^508^, ^509^ ^]^ DHI,^[^ ^418^, ^510^, ^511^ ^]^ DHICA,^[^ ^417^ ^]^ DHI and DHICA^[^ ^419^ ^]^), and cuttlefish melanins (*Sepia officinalis* ^[^ ^201^, ^512^ ^]^), often in combination with computational studies.

Atomic force microscopy (AFM)^[^ ^513^ ^]^ is a form of scanning probe microscopy capable of generating high resolution images (down to the Å scale) using tips with various functionalities. AFM studies typically show melanin particle aggregates (≈100–200 nm) of smaller particles (≈1–10 nm) that either assemble into larger particles in natural samples, or are deposited as thin films on substrates for more applied studies. AFM has been used to analyze melanins from a variety of sources including: synthetic melanins (e.g., based on l‐DOPA,^[^ ^514^, ^515^, ^516^, ^517^, ^518^ ^]^ DHI,^[^ ^229^, ^519^ ^]^ DHICA,^[^ ^520^ ^]^ DHI and DHICA,^[^ ^521^ ^]^ DHN^[^ ^522^ ^]^); *Nigella sativa*;^[^ ^412^ ^]^ fungi (Aspergillus fumigatus^[^ ^484^ ^]^); cuttlefish (*Sepia officinalis* ^[^ ^201^, ^523^, ^524^ ^]^), various other cephalopods species (*Sepia esculenta*, *Sepia lycidas*, *Sepia pharaonis*, *Sepiella japonica*, *Euprymna berryi*, and *Uroteuthis* (*Photololigo*) *edulis* ^[^ ^525^ ^]^); feathers of black fish crows (*Corvus ossifragus*) and iridescent wild turkeys (*Melleagris gallopavo*),^[^ ^524^ ^]^ black human hair,^[^ ^524^ ^]^ human neuromelanin,^[^ ^490^ ^]^ and human eye melanosomes.^[^ ^189^, ^526^ ^]^ Such studies have also enabled the elucidation of features such as the shape and size of melanosomes (e.g., in black hair are ellipsoidal eumelanosomes, whereas those in red hair are mainly spherical pheomelanosomes^[^ ^527^ ^]^), and the presence of cosmetic residues on the surface of hair.^[^ ^528^ ^]^ Force‐indentation measurements have revealed different mechanical properties of retinal pigment epithelium melanosomes isolated from human donors that may be related to the presence of thin deposits of lipofuscin on the surface of the melanosomes,^[^ ^529^ ^]^ electrostatic force microscopy and conductive‐AFM were used to spatially resolve the electrical properties of synthetic melanins (e.g., based on l‐DOPA^[^ ^521^, ^530^ ^]^), cuttlefish (*Sepia officinalis* ^[^ ^512^ ^]^) and magnetic force microscopy has been used to examine the magnetic properties of melanin–Fe_3_O_4_ nanoparticles.^[^ ^511^ ^]^

### 2.27 Computational Studies

Computational studies (i.e., gold biotechnology), facilitated by the rapid growth in processing power over the last few decades, have driven a dramatic expansion in the use of computer simulation leading some commentators to suggest that simulation has joined experiment and theory as one of the key pillars of science.^[^ ^355^, ^531^, ^532^, ^533^, ^534^, ^535^, ^536^, ^537^, ^538^ ^]^ The multiscale modeling approach describes a range of different simulation techniques that are applicable to the study of systems spanning the full spectrum of time and length scales. At the very largest end computer simulation can be used to study Galaxies and the Universe, while at the opposite end atomistic simulation can be used to understand how the macroscopic properties of systems are related to their underlying atomic structure. These atomistic techniques have been widely used to complement experimental studies on the biology underpinning melanogenesis, structure–property relationships of the melanins produced and organisms/tissues containing melanins.

Atomistic simulation approaches fall broadly into two categories, electronic structure methods, such as density functional theory (DFT), and classical molecular dynamics (cMD) that involve a system of atoms in time according to Newton's equations of motion where the forces are determined from empirically derived force fields. Electronic structure calculations allow determination of the fundamental gap between the highest occupied molecular orbital (HOMO) and lowest unoccupied molecular orbital (LUMO) that, to a first order approximation, represents the minimum excitation energy. Despite limitations due to the number of atoms that can be studied and theoretical approaches, early studies suggested that small oxidative and tautomeric differences can result in significantly different fundamental gaps for eumelanin.^[^ ^539^, ^540^ ^]^ These calculations support the theory that eumelanin is an ensemble of different chemical species, helping to explain the monotonic broad‐band UV–vis absorption spectra.^[^ ^541^ ^]^ More recent simulations involving larger numbers of atoms suggest that geometric disorder alone is also able to explain the UV–vis spectra for eumelanin.^[^ ^542^ ^]^ The ability to predict how the addition of functional groups alters the energy of the HOMO and/or LUMO using simulation allows prediction of absorption/emission spectra (see, for example, ref. ^[^ ^543^ ^]^) for devices such as biocompatible semiconductors.^[^ ^544^ ^]^ In addition to enabling the simulation of optical properties, electronic structure simulations can be employed to calculate the thermodynamics and kinetics of these processes.^[^ ^545^ ^]^

**Figure 7:**
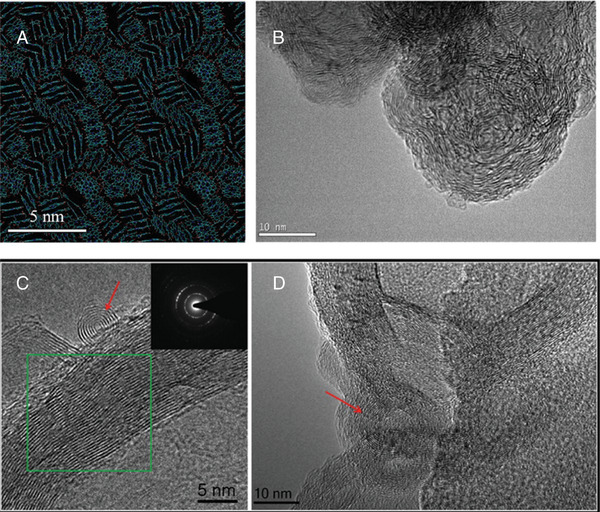
A) Snapshot of the simulated aggregate made of 375 IMIM tetramers at the steady state of self‐assembly. B) Typical TEM image of eumelanin produced from the oxidation of dopamine (2 g L^−1^ in an aerated Tris buffer, at 50 × 10^−3^ m and at pH 8.5, reaction time of 24 h). C,D) High‐resolution TEM images of eumelanin on other locations of the TEM grid. The inset of panel C shows an SAED pattern taken from the green‐boxed region. The red arrow in panels C and D indicates that the molecules aggregate and form an onion‐like nanostructure composed of stacked planes arranged in concentric rings. Reproduced with permission.^[^ ^548^ ^]^ Copyright 2013, American Chemical Society.

As mentioned above the major weakness associated with electronic structure simulations in their computational expense that limits them to the study of relatively small systems. Unfortunately, many processes occur on length scales beyond the reach of DFT.^[^ ^546^ ^]^ By neglecting electronic information cMD simulations enable the study of millions of atoms enabling the study of processes such as polymerization^[^ ^547^ ^]^ and self assembly (**Figure** **7**),^[^ ^548^, ^549^ ^]^ and moreover, to examine the kinetic and transport properties in enzyme structures.^[^ ^550^ ^]^

Bioinformatic studies have been used to examine functional and structural genomics (genetics,^[^ ^551^, ^552^ ^]^ gene expression of tyrosinase‐induced melanogenesis,^[^ ^553^ ^]^ albinism‐associated single nucleotide polymorphisms reported in oculocutaneous albinism,^[^ ^554^, ^555^, ^556^, ^557^ ^]^ identification of potential inhibitors against Rab38 and melanoma cancer^[^ ^558^ ^]^), proteomics (protein conformations and interactions (e.g., melanin‐concentrating hormone;^[^ ^559^, ^560^, ^561^, ^562^, ^563^, ^564^ ^]^ melanin‐concentrating hormone receptors^[^ ^563^, ^565^, ^566^, ^567^ ^]^ and their antagonists;^[^ ^563^, ^566^, ^568^, ^569^, ^570^ ^]^ structure–function relationships of tyrosinase mutants,^[^ ^571^, ^572^, ^573^, ^574^ ^]^ substances that inhibit tyrosinase activity;^[^ ^550^, ^575^, ^576^, ^577^, ^578^, ^579^, ^580^, ^581^, ^582^, ^583^, ^584^, ^585^, ^586^, ^587^, ^588^, ^589^, ^590^, ^591^, ^592^, ^593^, ^594^, ^595^, ^596^, ^597^, ^598^, ^599^ ^]^ the role of melanocortin 1 Receptor (MC1R) in skin tanning with potential to resolve pigmentary disorders,^[^ ^600^ ^]^ physiology,^[^ ^601^, ^602^, ^603^, ^604^, ^605^ ^]^ and pathology.^[^ ^94^, ^606^, ^607^, ^608^ ^]^ Such studies can offer insight into intermolecular interactions with melanins,^[^ ^609^, ^610^, ^611^ ^]^ drug pharmacokinetics,^[^ ^94^, ^612^, ^613^, ^614^ ^]^ and antibody targeting for anticancer treatments.^[^ ^615^, ^616^ ^]^

## 3 Melanins for a Sustainable Future

Melanins (and analogues thereof) have the potential for involvement in each of the different industry sectors across the world:^[^ ^30^, ^424^, ^426^, ^617^, ^618^, ^619^, ^620^, ^621^, ^622^ ^]^ their production, extraction, and purification (e.g., agricultural/fermentation/insect/marine sources, potentially involving blue/brown/gray/green/white/yellow biotechnologies)^[^ ^619^, ^623^ ^]^ is dealt with by the primary economic sector; their use in manufacturing products (e.g., electronics, plastics, textiles, potentially involving red/white/yellow biotechnologies)^[^ ^619^, ^624^ ^]^ is dealt with by the secondary economic sector; the tertiary sector involves services (e.g., distribution of melanin‐containing products, healthcare (e.g., diagnosis/therapy), appropriate waste management/recycling of melanin‐containing products, potentially involving red/white/yellow biotechnologies); the quaternary sector (e.g., education (by using melanin in interdisciplinary teaching in higher education), research and development in academic/industry settings, potentially involving all types of biotechnologies); and the quinary sector, specialized services delivered by the highest level of government/industry decision/policy makers (potentially involving purple/violet biotechnologies), such as utilization of natural resources such as melanins to demonstrate commitment to corporate social responsibilities, or to achieve the UN SDGs (potentially involving all forms of biotechnologies). The 17 UN SDGs aim to end poverty, protect the planet, and ensure all people enjoy peace and prosperity by 2030. The SDGs are complex real world problems, and melanins have the potential to play a role in multidisciplinary, interdisciplinary, and transdisciplinary solutions to these challenges. The SDGs and some examples of the potential involvement of melanins in their solution are summarized in **Table** **3**, and examples of potential biotechnological applications of melanins and melanin‐based materials are summarized in **Table** **4**.

**Table 3:** The UN SDGs and some examples of the potential involvement of melanins in their solution

UN SDG	Examples of potential solution involving melanins
1) End poverty	Creation of jobs involving melanin in each of the industry sectors.^[^ ^730^, ^731^ ^]^
2) Zero hunger	Improved agricultural processes (e.g., employing novel agrochemicals and chemical biology techniques, resulting in higher crop yields, nutritional value, and food security) for melanin containing foods.^[^ ^730^ ^]^
3) Global health and well‐being	Production of affordable medications and/or materials for medical interventions employing melanins [diagnostics,^[^ ^732^ ^]^ drug delivery devices,^[^ ^733^ ^]^ sensors,^[^ ^734^, ^735^, ^736^, ^737^ ^]^ theranostic agents,^[^ ^269^, ^276^, ^277^, ^278^, ^279^, ^286^, ^628^, ^738^, ^739^ ^]^ and tissue scaffolds.^[^ ^505^, ^740^ ^]^
4) Quality education	Development and delivery of affordable, accessible, and inclusive educational resources involving melanins in multidisciplinary, interdisciplinary, and transdisciplinary teaching activities (e.g., in the further/higher education contexts).^[^ ^741^, ^742^, ^743^, ^744^, ^745^ ^]^
5) Gender equality	Achieving equality of representation and salaries for all gender identities across the industries involving melanins, as well as negation or reduction of labor intensive traditional gender roles (e.g., waste management), thereby improving opportunities.^[^ ^746^ ^]^
6) Clean water and sanitation	Development of water purification processes involving melanins.^[^ ^397^, ^648^, ^747^, ^748^, ^749^ ^]^
7) Affordable and clean energy	Development of green affordable, reliable, and sustainable melanin‐based materials for energy harvesting, storage and use (e.g., batteries,^[^ ^393^ ^]^ capacitors,^[^ ^661^, ^676^ ^]^ switches^[^ ^512^ ^]^), with a circular economy perspective.^[^ ^648^ ^]^
8) Decent work and economic growth	Creation of jobs involving melanins in each of the industry sectors across the globe.^[^ ^648^, ^649^, ^731^, ^742^, ^750^ ^]^
9) Industry, innovation and infrastructure	Supporting entrepreneurial/inventive/innovative jobs involving melanins, and research and development of melanin containing materials, processes, products, and technologies.^[^ ^649^, ^742^, ^750^ ^]^
10) Reduced inequalities	Achieving equality of representation and salaries for all diversity groups across the industries involving melanins; reduce inequality between countries, and thereby enhance global security.^[^ ^745^, ^749^, ^751^ ^]^
11) Sustainable cities and communities	Development of cities/communities utilizing sustainable sources of energy, food, housing, transport, water, etc. (all of which could involve melanins in some way).^[^ ^649^ ^]^
12) Responsible consumption and production	Responsible consumption and production of goods containing melanins integrated within a circular economy^[^ ^648^, ^649^, ^731^, ^750^ ^]^
13) Climate action	Significant reductions in energy consumption to generate melanin‐based electronics relative to inorganic‐based electronics.^[^ ^731^, ^752^, ^753^ ^]^
14) Life below water	Developing materials and methods to conserve and cultivate oceans, seas, and marine resources to provide sources of melanins (e.g., minimizing/eradicating pollution from manufacturing/distribution, and the development of degradable melanin‐based materials for packaging).^[^ ^750^ ^]^
15) Life on land	Development of melanin‐based chemicals/materials/methods to ensure the sustainability of terrestrial biodiversity and ecosystems (and potentially also to provide sources of melanins).^[^ ^648^ ^]^
16) Peace, justice, and strong institutions	Promotion of effective, equitable, inclusive, and accountable institutions/societies committed to use of biorenewable resources such as melanins.^[^ ^754^, ^755^ ^]^
17) Partnership for the goals	Implementing and supporting global partnerships between researchers in the public, private and third sector to facilitate sustainable development involving biorenewable resources such as melanins.^[^ ^649^, ^730^, ^731^ ^]^

**Table 4:** Examples of biotechnological applications of melanins

Biotechnology classification	Potential applications of melanins
Blue biotechnology	Production, extraction, and purification of melanins from marine/sea bacteria, algae, fish, etc. used to produce feedstocks or materials for various applications.
Brown biotechnology	Production, extraction, and purification of melanins from bacteria, fungi, plants, etc. Management of Arid Lands and Deserts: innovation/creation of biotechnologies to enable/manage agriculture in arid lands and deserts.
Dark biotechnology	Defense‐related technologies, e.g., melanin‐based biosensors for microorganisms and toxins to cause diseases/death in humans, livestock, and crops; remediation of nuclear contamination.
Gold biotechnology	Computational studies devoted to study melanins and melanin‐based materials (e.g., bond conjugation and connectivity, gene expression, polymerization kinetics, drug binding, electronic properties, etc.).
Gray biotechnology	Production, extraction and purification of melanins from environmental sources (e.g., algae, bacteria, fish, plants, etc.) and their use for environmental applications (e.g., maintenance of biodiversity and the remediation of pollutants and/or nuclear contamination/waste).
Green biotechnology	Production, extraction and purification of agricultural process‐derived melanins from algae, bacteria, fungi, plants, of natural and transgenic varieties used to produce feedstocks or materials for various applications.
Purple/violet biotechnology	Bioethical issues (e.g., genetic modification of organisms to produce melanins), intellectual property (e.g., patents on production/applications of melanins and melanin containing products), publications regarding biotechnology involving melanins (academic and gray literature (e.g., white papers), safety practice and safety studies of law (e.g., regulations of melanin containing products and their safety).
Red biotechnology	Melanin‐based materials/products employed in medical, pharmaceutical and health applications (e.g., biodegradable power sources, biosensors, drug delivery devices, electrodes for neuromodulation, imaging/theranostics, tissue engineering).
White biotechnology	Indistrially viable production, extraction, and purification of melanins produced by fermentation of bacteria/yeast, or enzyme‐mediated synthesis of melanins from various feedstock monomers; and development and production/processing of melanins and melanin‐based materials.
Yellow biotechnology	Production, extraction, and purification of melanins from insects. Melanin‐based biotechnology to control insects (e.g., sensors and devices for controlled release of bioactives).

**Figure 8:**
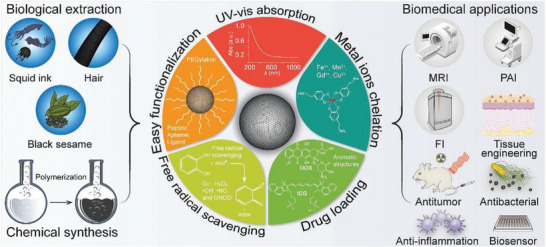
Schematic illustration of the synthesis, property, and biomedical applications of multifunctional melanin nanoparticles (MelNPs). Reproduced with permission.^[^ ^625^ ^]^ Copyright 2019, Wiley.

As outlined above, we foresee significant potential for melanins (and analogues thereof) throughout various sectors of the economy.^[^ ^426^, ^617^, ^618^, ^619^ ^]^ The variety of analytical techniques available to interrogate melanins and materials/organisms containing melanins has yielded a significant body of literature on their properties that enable a variety of high value biomedical applications (summarized in a number of excellent reviews,^[^ ^47^, ^618^, ^625^, ^626^, ^627^, ^628^, ^629^, ^630^ ^]^ see **Figure** **8** ^[^ ^625^ ^]^), demonstrating their potential for the healthcare industry in achieving the UN SDGs.^[^ ^631^ ^]^

There is also significant potential for melanins as sustainably sourced feedstocks/materials for technical applications, for example, as coatings,^[^ ^37^ ^]^ as dyes for textiles,^[^ ^624^, ^632^, ^633^, ^634^, ^635^, ^636^ ^]^ for application in environmental remediation (e.g., heavy metal, nuclear contamination/waste, etc.),^[^ ^629^, ^637^ ^]^ and as sustainable components of electronic products (as emphasized in Figure 6) due to their sustainable nature and prospects for biodegradation at the end of their useful lifetime.^[^ ^37^, ^424^, ^426^, ^503^, ^617^, ^618^, ^619^, ^620^, ^638^, ^639^, ^640^, ^641^ ^]^ It is noteworthy that in 2017, 94 billion tonnes of resources were extracted worldwide and this is forecast to rise to 184 billion tonnes by 2050, which is 400% more than the Earths capacity.^[^ ^642^ ^]^ This overextraction and the associated practices cannot be sustained. For instance, current extractive practices can contribute to environmental degradation through the creation of greenhouse gases, resource scarcity can trigger price volatility, reputational capital comes under scrutiny as investors, businesses, consumers, and other stakeholders demand more ecological products and services; moreover, businesses are subject to further risks as local/national/global policies adapt and change to include higher taxes on CO_2_ emissions and contribute to the continuation and recreation of social inequalities.^[^ ^642^, ^643^, ^644^ ^]^

**Figure 9:**
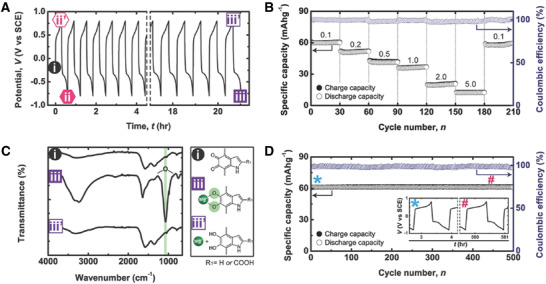
Charge–discharge and Coulombic efficiencies of NatMel. A) Galvanostatic charge–discharge cycles of NatMel electrodes are shown in 0.5 m Mg(NO_3_)_2_ with the current density of ±1.0 A g^−1^. Cycles (1–10) are displayed from total 30 cycles. B) NatMel shows the stable capacities of 61.6 ± 0.3 mA h g^−1^ from current density of 0.1 A g^−1^ while retaining 60.8 ± 0.7 mA h g^−1^ even after 150 cycles at the various current densities. C) FT‐IR spectra of NatMel are measured for (i) pristine, (iii) discharged, and (iii′) charged after 30 discharge–charge cycles. The transient and distinctive stretch at 1080 cm^−1^ suggests that ether bonds are formed and disrupted during (dis)charge cycles. D) Reversible charge–discharge cycles (500 cycles) of NatMel cathodes were measured in 0.5 m Mg(NO_3_)_2_ with the current density of ±0.1 Ag^−1^. NatMel cathodes exhibit stable specific capacities of >61.3 ± 0.8 mA h g^−1^ after 500 cycles. Coulombic efficiencies are maintained in the range of >99.2%. Insets represent the curves from initial (*) and final (#) charge–discharge cycles. Reproduced with permission.^[^ ^657^ ^]^ Copyright 2014, Wiley.

**Figure 10:**
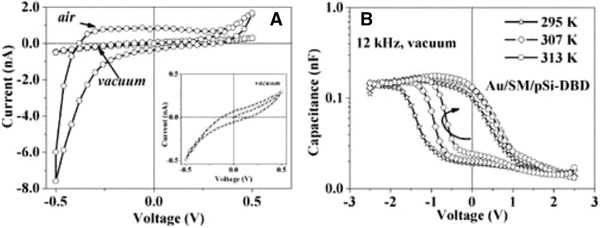
a) Current–voltage hysteresis loops of a melanin‐based device on ITO/glass support (Au/SM/ITO/glass) in air and under vacuum (*p* = 10^−5^ mbar). The inset shows the magnified loop collected under vacuum. b) *C*–*V* hysteresis loops collected under vacuum at different temperatures, starting from room temperature 295 K, for a melanin‐based MIS device on pSi (Au/SM/pSi‐DBD) at a sine wave voltage frequency of 12 kHz. Reproduced with permission.^[^ ^665^ ^]^ Copyright 2011, Wiley.

**Figure 11:**
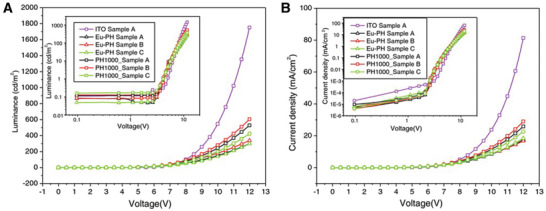
A) Luminance and B) current density measured for the OLEDs equipped with the different anodes. ‐□‐, cyan: ITO; ‐△‐, green, red, black: Eu–H; ‐□‐ green, red, black: PH1000. Reproduced with permission.^[^ ^669^ ^]^ Copyright 2016, Wiley.

Since 2007 the Circular Economy (CE) agenda has rapidly mobilized in business and policy spheres^[^ ^645^, ^646^, ^647^, ^648^, ^649^ ^]^ highlighting opportunities for a new economy that is “restorative and regenerative by design.” The principles of the CE aim to design out waste and harmful materials, and keep goods at their highest utility and in circulation for as long as possible.^[^ ^643^ ^]^ The CE discourse holds promise and new occasions to innovate, with electronic and bio‐based electronic technologies being one such area. Electronic devices are ubiquitous (touching every aspect of our lives from birth to death) and underpinning the economic success of countries across the world. Advances in the manufacturing and miniaturization of electronics (transistors, microprocessors, telecommunications, computers, etc.) during the 3rd industrial revolution enabled the 4th industrial revolution (particularly additive manufacturing, cyber physical systems and biotechnology).^[^ ^650^ ^]^ Different components of electronic technologies employ conductors and semiconductors to fulfil specific roles within the devices being manufactured, with organic conductors/semiconductors playing an increasingly prominent role in electronic devices (e.g., in flexible displays, wearable electronics, etc.). Organic conductors and semiconductors (e.g., derivatives of carbon nanotubes, graphene, poly(3,4‐ethylenedioxythiophene), etc.) are produced using a variety of chemical methodologies (e.g., solution or vapour phase synthesis) on a vast scale, however, their green synthesis from renewable resources,^[^ ^651^ ^]^ or replacement with natural conducting or semiconducting polymers such as melanins has not yet been explored fully.^[^ ^407^, ^425^, ^620^, ^652^, ^653^, ^654^ ^]^ However, proof of concept has been shown for the application of melanins in a variety of common electronic components, including (but not limited to): batteries^[^ ^393^, ^655^, ^656^, ^657^, ^658^, ^659^, ^660^ ^]^ (e.g., natural *Sepia officinalis* melanin‐based batteries, see **Figure** **9**),^[^ ^657^ ^]^ capacitors^[^ ^661^, ^662^, ^663^, ^664^, ^665^, ^666^, ^667^ ^]^ (e.g., synthetic melanin‐based capacitors, see **Figure** **10**),^[^ ^665^ ^]^ light emitting diodes^[^ ^424^, ^668^, ^669^, ^670^ ^]^ (e.g., synthetic melanin‐inspired DHI/polystyrene sulfonate‐based LEDs, see **Figure** **11**),^[^ ^669^ ^]^ memory,^[^ ^424^, ^671^ ^]^ photoelectrodes for solar water splitting,^[^ ^672^, ^673^ ^]^ solar cells,^[^ ^674^, ^675^, ^676^ ^]^ and transistors.^[^ ^424^, ^425^, ^677^, ^678^, ^679^, ^680^, ^681^ ^]^

It is noteworthy that more than 50 million tonnes of electronic waste are discarded annually, with growth of ≈2 million tonnes a year, worth an estimated $57B USD in raw materials alone which gives an indication of the scale and possibility for innovation.^[^ ^682^ ^]^ If these current products are not dismantled and recycled in a safe manner, they pose ecological risks to the natural environment and occupational health risks to those involved in their disposal.^[^ ^683^ ^]^ For example, computers can contain up to 1000 chemicals^[^ ^684^ ^]^ including toxic substances such as arsenic, cadmium, lead, mercury, and phosphorous.^[^ ^685^ ^]^ Workers exposed to these chemicals run the risks to their health including blood poisoning, respiratory illness, endocrine failure, infertility, and damage to major organs.^[^ ^686^ ^]^ With the waste sector being the third largest employer worldwide,^[^ ^687^, ^688^ ^]^ the shift to biotechnological routes to production of electronics has significant potential for both beneficial and unintended economic and societal impacts.

Organic electronics (OEs), if produced at scale, are cost effective to manufacture compared with metal oxide semiconductor processes due to a reduction in vacuum processing temperature (OE at ≈115 °C whereas CMOS at ≈1000 °C), thereby reducing energy consumption, employing lower cost materials, and potentially roll‐to‐roll fabrication methods to print/coat/laminate or embed material onto substrates.^[^ ^617^, ^689^, ^690^ ^]^ There is consequently potential for greener manufacture processes which produce less toxic materials and by‐products that have promise in medical and technical applications (e.g., biosensors,^[^ ^691^ ^]^ printing^[^ ^692^, ^693^, ^694^, ^695^, ^696^, ^697^ ^]^), especially given that OEs are comparatively lightweight and flexible.^[^ ^617^, ^620^, ^690^ ^]^ Further research into OEs has the potential to reduce environmental impacts by adoption of renewable resources and green processes, thereby effectively managing the amount of e‐waste generated annually (currently ≈50 million tonnes);^[^ ^640^, ^698^, ^699^, ^700^, ^701^, ^702^, ^703^ ^]^ and health impacts by use of the technologies for medical applications. OE development is not without challenges^[^ ^704^, ^705^, ^706^ ^]^ and requires a market demand to be articulated^[^ ^707^, ^708^ ^]^ to create scalability and realize the potential economic and environmental benefits.

One of the barriers to the development of OE employing melanin‐based components is that in contrast to naturally occurring biopolymers such as polynucleic acids (e.g., DNA/RNA) or polyamides (e.g., peptides/proteins) which have specific sequences of monomers and therefore reproducible properties if appropriately purified; melanins are a class of biopolymers which do not have a specific sequence of monomers and therefore their properties are not necessarily reproducible (akin to polysaccharides such as cellulose), however, this does not necessarily preclude their use in real world applications (e.g., as dyes for textiles). Potential solutions to this include careful cultivation of melanin producing species under controlled conditions (e.g., employing expertise in blue, brown, gray, green, white, and yellow biotechnology), wherein the environment is controlled (e.g., defined media for industrial scale fermentations of bacteria/yeast or industrial cultivation of cuttlefish/fungi), or indeed the development of melanin‐inspired synthetic analogues (e.g., polydopamine);^[^ ^654^, ^709^, ^710^, ^711^, ^712^ ^]^ and balancing the necessity for high levels of reproducibility (e.g., biosensors for biomedical applications, electronics for long term biomedical/technical applications) with utility (e.g., dyes, components of degradable/transient electronics (e.g., batteries)).

In terms of research and development and production, Asia and the USA lead for display technologies and robotics, with Europe leading in fundamental material development.^[^ ^713^, ^714^ ^]^ There is potential to invest in and/or share infrastructures associated with manufacture (potentially reducing inequality between countries).^[^ ^715^ ^]^ The further development of regulatory frameworks regarding electronics production are also needed, contemplating the lifecycle of the electronic devices (from design to production, substitution of toxic materials,^[^ ^716^, ^717^, ^718^, ^719^, ^720^, ^721^, ^722^ ^]^ durability, efficiency,^[^ ^723^, ^724^ ^]^ and disposability^[^ ^620^, ^717^ ^]^), especially with regard to the impact this could have on those operating in the waste sector (recycling feedstocks could diminish if the electronics are transient/degradable, impacting livelihoods of those in the waste sector^[^ ^725^ ^]^). A transdisciplinary research agenda is required that not only bridges the academic practitioner gap, but also enables the translation of scientific findings into concrete contexts^[^ ^726^ ^]^ to ensure future advances, such as the use of melanins in biosensors for medical applications, taking into account wider implications including economics (markets and demands), legislation (patents and frameworks), and ensuring the production and consumption practices minimize the impact on the environment, society as well assessing the ethical implications,^[^ ^727^ ^]^ the latter of which (legislation/ethics) being purple/violet biotechnologies. These types of research are complex^[^ ^728^ ^]^ but adopting such an approach will help to ensure that a circular economy by design can be regenerative, restorative, and inclusive of people and help achieve the UN SDGs.

## 4 Conclusion

As highlighted throughout the review, melanins are a class of biopolymers with diverse origins, chemical compositions, and functions that are widespread in nature. Their abundance, chemical/electrical/optical/paramagnetic properties offer them significant potential for application in materials science and engineering for a multitude of technical and biomedical applications. This review offers readers an overview of the analytical techniques commonly used to study melanins from various sources including agriculture, fermentation, insects, marine sources, etc., which potentially involve a spectrum of biotechnologies (blue, brown, gray, green, white or yellow). The analytical techniques can be used in a variety of disciplinary contexts for multidisciplinary, interdisciplinary, and transdisciplinary research and development (confirmed by the breadth of disciplinary backgrounds of the authors). We believe that melanins have significant potential for use as sustainable resources for advanced biotechnological applications (e.g., red biotechnology, biomedical applications), and that they may facilitate our achievement of the UN SDGs through engagement with the UN's Six PRME initiative.

## Conflict of Interest

The authors declare no conflict of interest.

## Author Contributions

All authors contributed to the conceptualization of this paper. H.G. and J.G.H. prepared the original draft. All authors reviewed and edited this paper. R.L.M, A.M.T., and J.G.H. supervised the work. J.G.H. headed the project administration. H.G., A.S., H.L., S.T.M., P.L.M.‐H., A.M.T., R.L.M, and J.G.H. managed funding acquisition.
